# Importance of Weight Loss Maintenance and Risk Prediction in the Prevention of Type 2 Diabetes: Analysis of European Diabetes Prevention Study RCT

**DOI:** 10.1371/journal.pone.0057143

**Published:** 2013-02-25

**Authors:** Linda Penn, Martin White, Jaana Lindström, Annemieke Th. den Boer, Ellen Blaak, Johan G. Eriksson, Edith Feskens, Pirjo Ilanne-Parikka, Sirkka M. Keinänen-Kiukaanniemi, Mark Walker, John C. Mathers, Matti Uusitupa, Jaakko Tuomilehto

**Affiliations:** 1 Institute of Health and Society, Newcastle University, Newcastle upon Tyne, United Kingdom; 2 Department of Chronic Disease Prevention, Diabetes Prevention Unit, National Institute for Health and Welfare, Helsinki, Finland; 3 Department of Human NUTRIM School for Nutrition, Toxicology and Metabolism, Maastricht University Medical Center, Maastricht, The Netherlands; 4 Department of General Practice and Primary Health Care, University of Helsinki, Helsinki, Finland; 5 Central Hospital and Folkhälsan Research Center, Helsinki, Finland; 6 Division of Human Nutrition, Wageningen University, Wageningen, The Netherlands; 7 The Diabetes Center, Finnish Diabetes Association, Tampere, Finland; 8 Institute of Health Sciences (General Practice), University of Oulu and Central Hospital, Oulu, Finland; 9 Institute of Cellular Medicine (Diabetes), Newcastle University, Newcastle upon Tyne, United Kingdom; 10 Human Nutrition Research Centre, Institute for Ageing and Health, Newcastle University, Newcastle on Tyne, United Kingdom; 11 Institute of Public Health and Clinical Nutrition, University of Eastern Finland, Kuopio, Finland; 12 South Ostrobothnia Central Hospital, Seinäjoki, Finland; 13 Hospital Universitario La Paz, Madrid, Spain; 14 Centre for Vascular Prevention, Danube-University, Krems, Austria; Federal University of São Paulo (UNIFESP), Escola Paulista de Medicina, Brazil

## Abstract

**Background:**

Prevalence of type 2 diabetes (T2D) is increasing worldwide. T2D prevention by lifestyle intervention is effective. Pragmatic scalable interventions are needed, with evidence to efficiently target and monitor such interventions. We report pooled analyses of data from three European trial cohorts: to analyse T2D incidence, sustained weight loss and utility of risk predictors.

**Methods:**

We analysed data on 749 adults with impaired glucose tolerance (278 men and 471 women, mean age 56 years, mean BMI 31 kgm^−2^) recruited between 1993 and 2003, and randomised to intensive lifestyle intervention (I) or lifestyle advice control (C). The intervention aimed to increase physical activity, modify diet, and promote weight loss≥5%. Using Cox-regression survival analysis, we assessed T2D incidence and the impact on T2D incidence of sustained weight loss, and of baseline cut-point values of FINDRISC score, fasting plasma glucose (FPG), and HbA_1c_.

**Results:**

Mean follow-up duration was 3.1 years. T2D was diagnosed in 139 participants (I = 45/379, C = 94/370). Cumulative T2D incidence was 57% lower in the intervention compared with the control group (HR 0.42 (95% CI 0.29 to 0.60) P<0.001). Participants with ≥5% weight loss at one year had 65% lower T2D incidence (HR 0.35 (95% CI 0.22 to 0.56) P<0.001); maintaining ≥5% weight loss for two and three years further reduced T2D incidence. Recommended cut-points to identify those at high risk for T2D would have identified different proportions of European Diabetes Prevention Study (EDIPS) participants with similar hazard-ratios for intervention effect.

**Conclusions:**

Pooled analysis of EDIPS trial data reinforces evidence for T2D prevention by lifestyle intervention. Analysis showed the preventive effect of ≥5% weight loss, especially if maintained long term, which has utility for intervention monitoring. Analysis of proposed cut-points demonstrates difficulties in balancing risk and benefit, to efficiently target interventions and suggests evidence is needed to define clinical policy.

**Trial registrations:**

The Finnish Diabetes Prevention study, Helsinki, Finland: ClinicalTrials.gov; NCT00518167 The SLIM diabetes prevention study, Maastricht, The Netherlands: Clinical Trials.gov; NCT00381186 The EDIPS-Newcastle diabetes prevention study, Newcastle upon Tyne, UK: International Standard Randomised Controlled Trial Number; ISRCTN15670600.

## Introduction

Diabetes is predicted to affect 552 million people globally by 2030. [1] Type 2 diabetes (T2D) accounts for 90% of cases, resulting in substantial disability, premature mortality, and healthcare costs, [1] and its prevention is an important public health challenge. Genetic predisposition contributes to T2D risk, but disease development is strongly linked to excess body weight and lifestyle factors including diet and inactivity. [1] Both population level interventions to address obesity prevalence and interventions targeting individuals at high-risk of T2D are needed. [2]–[4] Impaired glucose tolerance (IGT) is a precursor of T2D that identifies individuals at high-risk [1].

The Finnish Diabetes Prevention Study (DPS) randomised controlled trial (RCT) demonstrated the efficacy of lifestyle intervention to prevent T2D in adults with IGT. [5] Subsequent RCTs have demonstrated efficacy in other populations. [2], [6] The European Diabetes Prevention Study (EDIPS) collaboration applied the DPS protocol in other European countries. The EDIPS trial cohorts comprised the DPS study in Finland; [5] the SLIM study in Maastricht, The Netherlands; [7] and the EDIPS-Newcastle study in Newcastle upon Tyne, UK. [8] All three EDIPS cohorts used a common protocol with similar intervention goals and study design, although some variability in local intervention delivery was permitted to enhance cultural acceptability. Details of the individual RCTs contributing to the pooled EDIPS data-set have been published [5], [7], [8].

A vital prerequisite in achieving reduction in T2D globally is to develop scalable, transferrable and cost-effective T2D prevention interventions that can be delivered in routine health care. [2], [9] The Finnish Development Programme for the Prevention and Care of Diabetes (DEHKO) and associated T2D prevention plan (FIN-D2D) is leading the way. [10], [11]. The UK National Institute for Health and Clinical Excellence (NICE) has published guidance for ‘Preventing type 2 diabetes: risk identification and interventions for individuals at high-risk’. [2], [4] The EDIPS RCTs recruited adults with IGT, but measuring glucose tolerance is impractical and too costly for large scale screening. [2] Instead, translational studies have used prospective risk scores, such as the Finnish Diabetes Risk Score (FINDRISC), [12] to identify high-risk individuals. However risk scores are population specific, provide valid individual risk estimates only when all data items are available and accurate, and cannot diagnose T2D. The NICE Programme Development Group for T2D prevention has advocated a two-stage screening strategy, using a risk score followed by a blood test for fasting plasma glucose (FPG) or glycated haemoglobin A_1c_ (HbA_1c_), to confirm high-risk. [2] This is a pragmatic strategy, but RCT evidence for effective and sustained T2D prevention [13], [14] is derived from at-risk populations identified by IGT and extrapolation to other high risk groups may not be warranted. In addition, many translational studies use weight loss at one year follow-up as the primary outcome. [15] The effect of achieving a weight loss goal at Year 1, and subsequent maintenance, has not been demonstrated. Percentage weight loss provides an easily-measured self-regulation goal for intervention participants [2] and the number of participants who achieve a pre-determined percentage weight loss could offer a useful key performance indicator for intervention monitoring.

The EDIPS RCTs aimed to evaluate the efficacy of similar lifestyle intervention in three European populations. In this paper, we report pooled analysis of data from the EDIPS cohorts. Weight loss ≥5% was an intervention goal [16] and, in this paper, we assessed the impact of sustained weight loss on T2D incidence. In addition, to inform future research and policy, we assessed the utility of alternative risk identification parameters and different cut point values in predicting outcomes in the EDIPS cohorts.

## Methods

The protocol for this trial and supporting CONSORT checklist are available as supporting information; see Checklist S1 and Protocol S1.

### Ethics Statement

Ethical approvals were given for the DPS by the ethics committee of the National Public Health Institute, Helsinki, Finland; for the SLIM study by the Medical Ethical Review Committee of Maastricht University; and for EDIPS-Newcastle by the Newcastle and North Tyneside NHS research ethics committee. All participants gave their written informed consent before starting the study.

### Trial Design

Details of the EDIPS protocol were similar in each country, have been published for each trial cohort [5], [7], [8] and are described briefly here.

### Randomisation and Masking

Participants were allocated at random in a 1∶1 ratio to an intensive lifestyle intervention to promote increased physical activity and dietary modification or to a minimal lifestyle advice control group. For the DPS and EDIPS-Newcastle (but not SLIM) the randomisation lists were generated and supplied by the coordinating centre in Helsinki and staff who made baseline measurements had no access to the randomisation lists. Neither staff delivering the intervention nor participants were masked to the study arm.

### Follow-up

Participants received a clinical assessment, including an Oral Glucose Tolerance Test (OGTT), anthropometric and blood biochemistry measurements, at baseline and annually thereafter. In addition they were asked to complete physical activity and dietary intake diaries. Measurement details, timelines and locations have been published. [5], [7], [8], [17] In March 2000, independent statistical analysis of the DPS data led to the end-point committee’s decision to end the DPS trial. Some participants completed six years in the study [5].

### Participants

We included adults over 40 years with BMI≥25 kgm^−2^ (or family history of T2D in the SLIM study) and with IGT, defined as plasma glucose concentration 7·8 to 11·0 mmoll^−1^ (140 to 199 mg/dl) two hours after a standard fasting Oral Glucose Tolerance Test (OGTT) (glucose load 75 g). [18] Exclusion criteria were diagnosis of T2D, chronic illness making participation in moderate physical activity impossible and medication (e.g. corticosteroids) that would compromise the intervention.

### Interventions

Intervention goals were based on a common protocol and comprised: weight reduction ≥5%, moderate physical activity for at least 30 minutes per day or equivalent (assessed as at least 30 minutes at level 5 MET/hours) and improved dietary quality, with increased fibre, reduced fat and reduced saturated fat intake. The DPS and EDIPS-Newcastle goals for fat intake were <30% total fat and <10% saturated fat, whilst the SLIM study goals were in accordance with the Dutch guidelines (Dutch Nutrition Council) that advised 30 to 35% total fat intake with <10% saturated fat [19].

Intervention delivery centred on individual counselling, including motivational interviewing. [20] Physical activity and food diaries, completed quarterly, facilitated personalised advice delivered by trained health professionals. Supervised physical activity sessions and access to leisure facilities were promoted.

### Control Condition

Control group participants were given brief written and verbal information about a healthy diet and the benefits of physical activity.

### Outcomes

The primary outcome was impact of lifestyle intervention on T2D incidence. Diagnosis of T2D was determined at annual follow-up using a standard OGTT with 2 hour venous plasma glucose ≥11·1 mmoll^−1^ or FPG≥7·8 mmoll^−1^ in accordance with WHO criteria. [18] In the DPS and EDIPS-Newcastle cohorts, a repeat measure confirmed the diagnosis and was a study end-point. In the SLIM cohort, diabetes diagnosis was based on a single OGTT. Secondary outcomes were weight loss, increased physical activity and improved dietary quality.

Sample size was determined for the DPS with 80% power and 95% significance to detect 30% reduction of T2D incidence in the intervention group compared with the control group. This calculation was based on the assumption that 28% of adults with IGT would progress to T2D in five years. The DPS, EDIPS-Newcastle and SLIM cohorts contributed to a planned total of 750 European participants [21].

### Data Analysis

For analysis of the pooled EDIPS data-set we used SPSS (IBM SPSS inc. Version 17). We used independent t-tests and Chi-squared tests to compare variables at baseline and Cox-regression survival analysis to assess risk of developing T2D, with T2D incidence as the explanatory variable controlling for trial cohort (Finland, The Netherlands, and The UK). For pooled analysis of the main trial outcome, we used RevMan (The Cochrane Collaboration Review Manager 5.1) to conduct meta-analysis assessing risk ratios using Mantel-Haenszel, random effects and Tau^2^ variance measure.

The EDIPS data-set includes cases with baseline FPG≥7·0 mmoll ^−1^ or HbA1c ≥6·5%. In 2012, these values would be in the diabetic range due to recent changes in WHO diagnostic criteria. [22], [23] We therefore analysed a sub-set of the EDIPS data with these cases removed as a sensitivity analysis.

For explanatory analysis of weight loss maintenance, we pooled intervention and control group data. In separate analyses, we compared those who achieved ≥5% weight loss at 12 months, those who achieved this goal at 12 months and maintained it at 24 months, and those who achieved this goal at 12 months, maintained it at 24 months and again at 36 months, with those who did not achieve these goals.

We analysed values of FINDRISC score, [12] FPG and HbA1c at baseline in relation to EDIPS outcomes. We used Cox-regression to analyse the effect of intervention on risk of developing T2D in EDIPS sub-sets defined by FPG and HbA1c cut-points recommended by NICE [22] and the American Diabetes Association (ADA) [24] and, for FPG, recommended by WHO [22] (the WHO expert group concluded that there was insufficient evidence to recommend HbA1c cut-points for non-diabetic hyperglycaemia [23]), as well as combinations of risk score and blood tests ranges.

## Results

### Incidence of T2D

**Figure 1:**
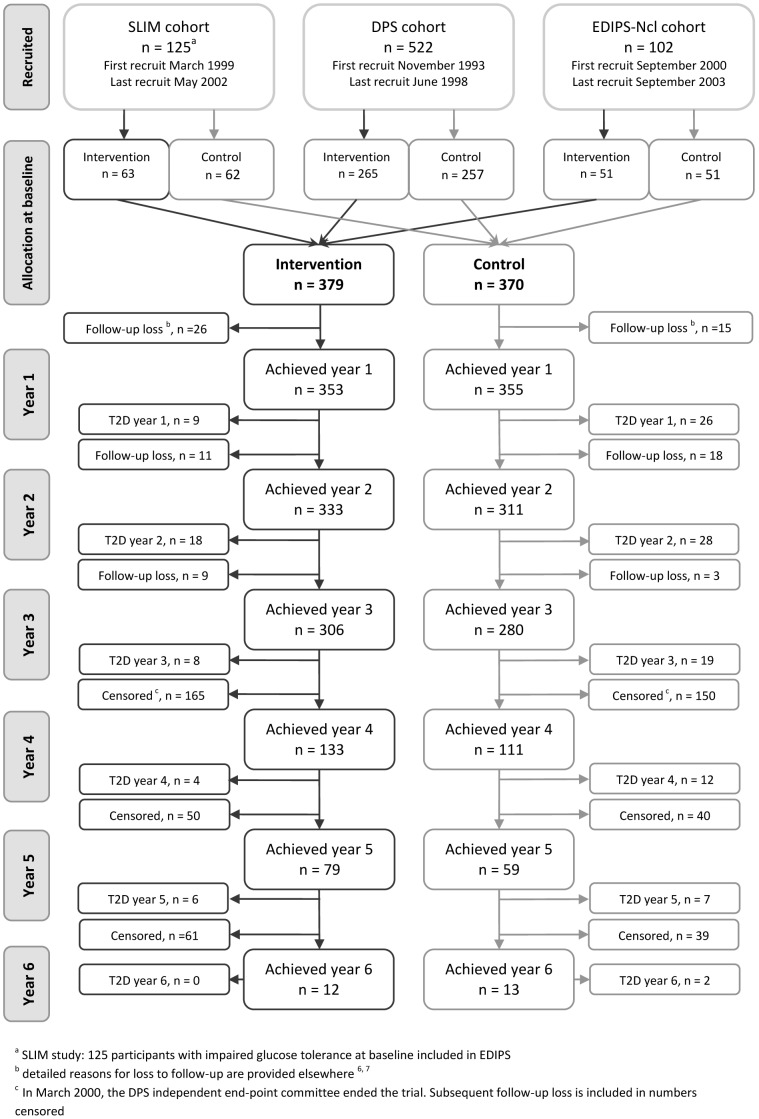
CONSORT Flow chart - recruitment by trial cohort and progress of participants. The flow chart refers to participants in the EDIPS study with impaired glucose tolerance at baseline.

**Figure 2:**
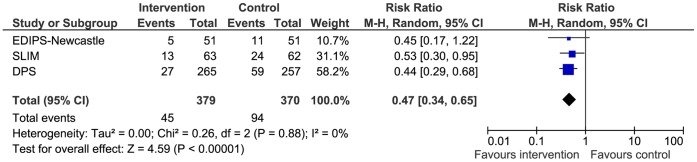
Forest plot comparison of the three studies contributing to the EDIPS data-set.

In the three European Diabetes Prevention Study (EDIPS) cohorts we recruited a total of 771 participants and followed them up for a mean duration of 3·1 years (maximum 6 years). [5], [7], [8] For the analyses presented here, we included 749 (DPS n = 522, SLIM n = 125, and EDIPS-Newcastle n = 102) with IGT at baseline (22 participants from the SLIM study with 2 hour plasma glucose value >11·0 moll^−1^ were excluded). Details of the recruitment dates, randomisation and trial progression are shown in Figure 1. Baseline characteristics included mean (SD) age 55·6 (8·1) years, BMI 31·2 (4·9) kgm^−2^, and FINDRISC score 14·0 (4·2). There were no statistical differences in any of the baseline measures between intervention and control groups (Table 1). T2D was diagnosed in 139 participants (I = 45, C = 94). The absolute incidence of T2D was 38·3 per 1000 person-years in the intervention group and 81·9 per 1000 person-years in the control group. On average, 7·4 persons were treated for a mean of 3·1 years to prevent one case of T2D (equivalent to a number needed to treat (NNT) of 22·9 for one year). In meta-analysis of pooled data from the three EDIPS study cohorts, there was no statistically significant heterogeneity between studies (Figure 2). Meta-analysis resulted in a pooled risk-ratio of 0·47 (95% CI 0.34–0.65).

**Table 1:** Baseline characteristics: mean (SD) for continuous variables and number (%) for categorical variables by trial arm.

	Intervention (n = 379)	Control (n = 370)
Continuous variables	Mean value (SD)	
Age (years)	55·4 (8·0)	55·7 (8·2)
BMI (kgm^−2^)	31·4 (5·0)	31·0 (4·8)
Waist (cm)	102·5 (11·2)	101·1 (10·4)
Hip (cm)	109·8 (10·3)	108·7 (10·8)
Weight (kg)	88·2 (14·8)	85·7 (13·6)
FINDRISC score^11^	14·2 (4·0)	14·0 (4·2)
Plasma glucose (mmol/l)		
* Fasting*	6·0 (0·8)	6·0 (0·7)
* 60 minute*	11·2 (2·0)^a^	11·1 (2·1)^b^
* 120 minute*	8·8 (1·5)	8·7 (1·4)
Plasma insulin (mU/l)		
* Fasting*	13·3 (7·8)^c^	12·8 (6·6)^d^
* 120 minute*	84·7 (61·0)^e^	83·4 (56·7)^f^
Blood pressure (systolic) (mmHg)	139 (17)	137 (16)
Blood pressure (diastolic)(mmHg)	84 (9)	85 (9)
HbA1c (%)	5·7 (0·5)^g^	5·7 (0·5)^h^
Cholesterol (mmoll^−1^ )	5·5 (1·1)^i^	5·5 (1·0)^j^
Triglycerides (mmoll^−1^ )	1·7 (0·9)^i^	1·7 (0·8)^j^
HDL Cholesterol (mmoll^−1^ )	1·2 (0·3)^i^	1·2 (0·3)^i^

**Categorical variables**	**Number (%)**	
FINDRISC^10^ categories		
Low <7	6 (1.7)	14 (3.9)
Slightly elevated 7–11	93 (25.8)	85 (23.9)
Moderate 12–14	93 (25.8)	96 (27.0)
High 15–20	147 (40.7)	142 (39.9)
Very high >20	22 (6.1)	19 (5.3)
Data unavailable	18 (4.7)	14 (3.7)
Sex		
* Male*	145 (38·3)	133 (35·9)
* Female*	234 (61·7)	237 (64·1)
Socio-economic status by educational level		
* Low*	109 (28·8)	111 (29·9)
* Medium*	97 (25·6)	92 (24·8)
* High*	100 (26·4)	100 (27·0)
* Data unavailable*	73 (19·3)	67 (18·3)
Smoking status		
* Non-smoker*	195 (51·5)	208 (56·1)
* Current smoker*	28 (7·4)	38 (10·2)
* Data unavailable*	156 (41·2)	124 (33·7)

a n = 348.

b n = 330.

c n = 354.

d n = 348.

e n = 351.

f n = 344.

g n = 370.

h n = 362.

I n = 375.

j n = 364.

**Figure 3:**
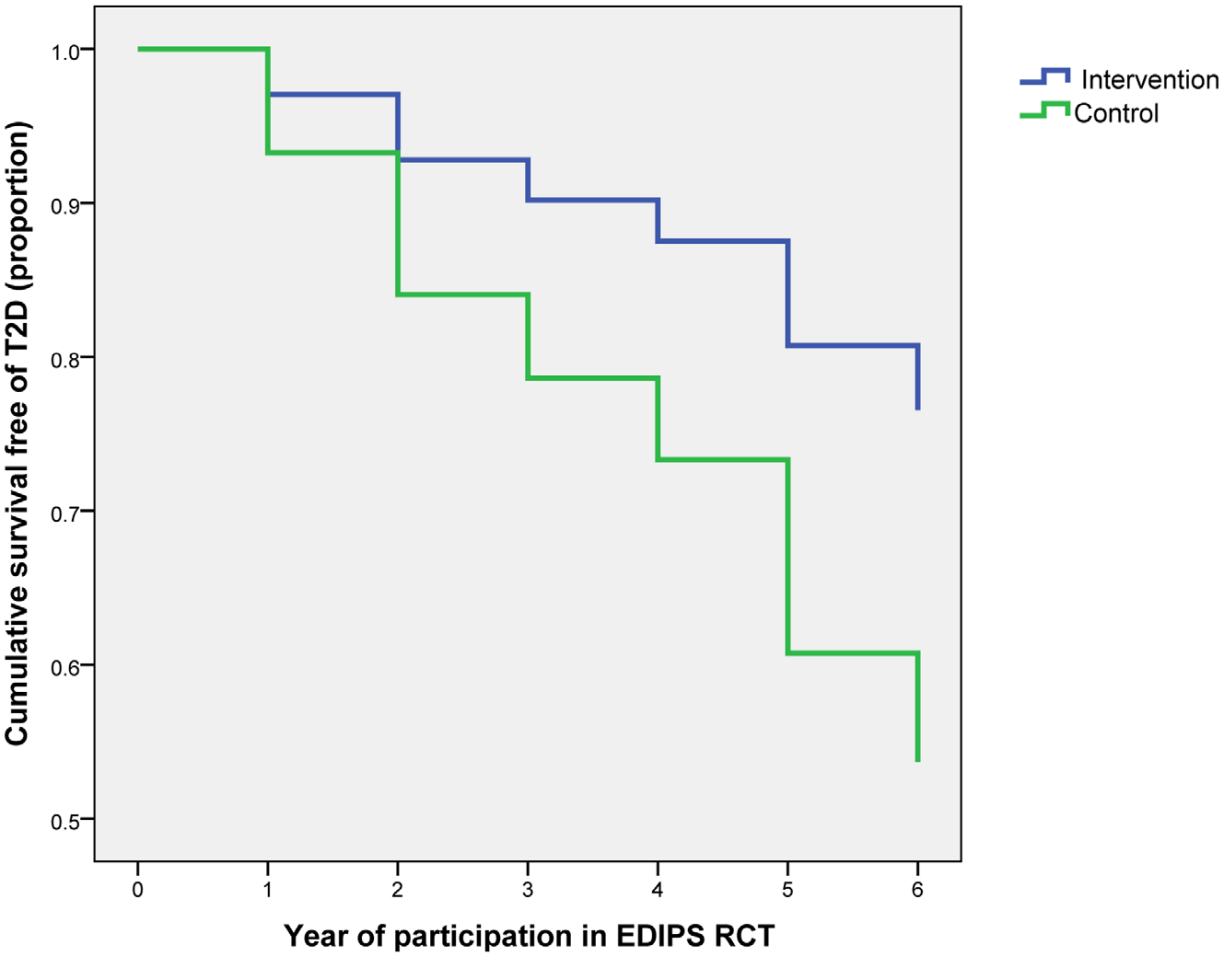
Progression to type 2 diabetes by trial arm.

Cox-regression analysis, controlling for trial cohort, suggested the cumulative incidence of T2D was 57% lower in the intervention than in the control group (hazard ratio 0·43 (95% CI 0·30 to 0·61); P<0·001) (Figure 3) (this differs from the risk ratio above (Figure 2), which does not account for survival). In sex-specific analyses, cumulative incidence of T2D was 63% lower for males (hazard ratio 0·37 (95% CI 0·21 to 0·65); P<0.001) and 52% lower for females (hazard ratio 0·48 (95% CI 0·30 to 0·77); P<0.001).

Cox-regression analysis of a sub-set (n = 632) in which cases having a single baseline value of FPG≥7·0 mmoll^−1^ or HbA_1c_ ≥6·5% were removed, [23] showed 62% reduction in T2D risk in the intervention group compared with the control group (hazard ratio 0·38 (95% CI 0·25 to 0·60), P<0·001).

### Impact of Weight Loss Maintenance

**Figure 4:**
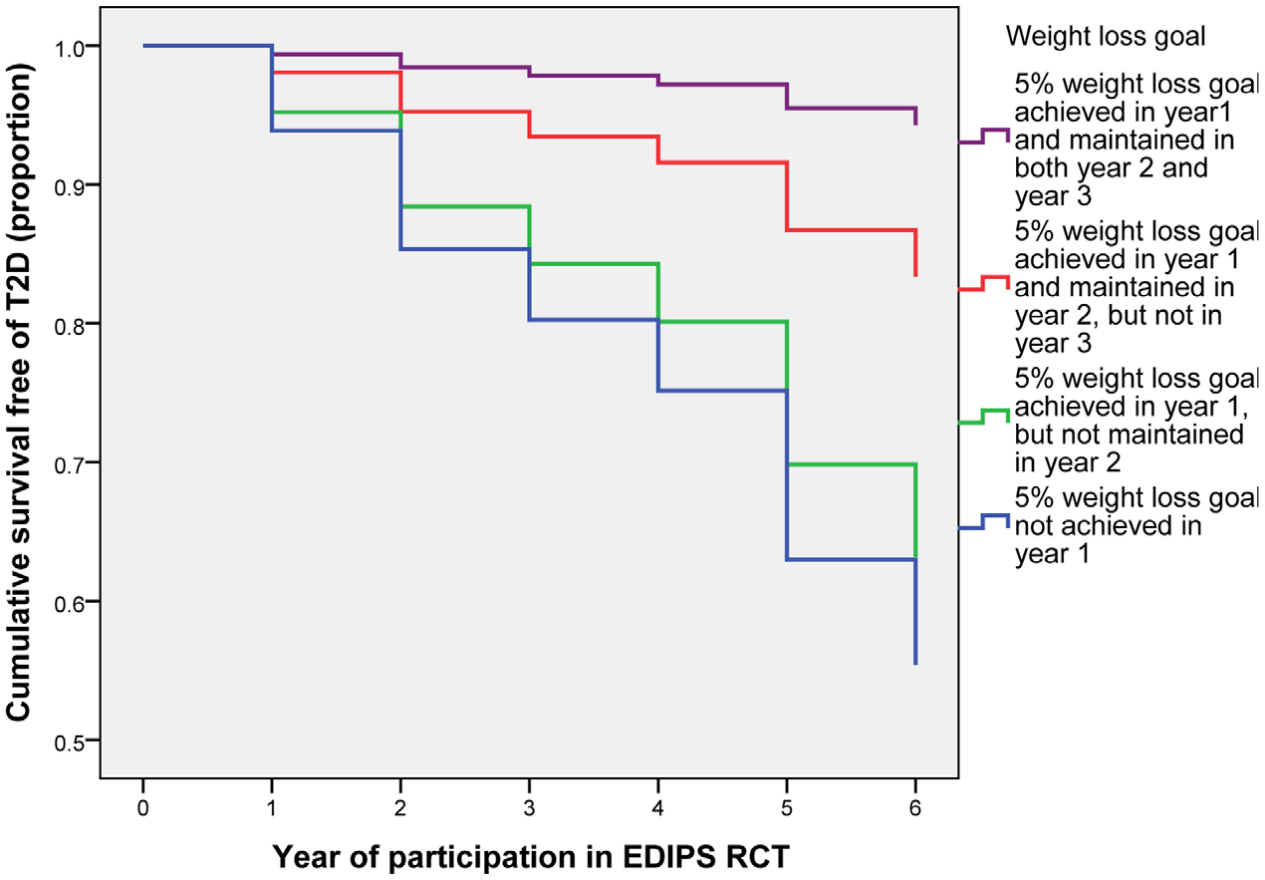
Progression to type 2 diabetes by weight loss achieved and maintained.

In the intervention group, 144 (38%) achieved ≥5% weight loss at Year 1, 105 (28%) maintained this at Year 2 and 86 (23%) at Year 3; whilst in the control group 50 (14%) achieved ≥5% weight loss at year one, 26 (7%) maintained this at year two, and 18 (5%) at year three (Chi-Squared tests, p<0·0001 for difference in the proportions achieving weight loss between intervention and control groups at each time point). Cox-regression analysis of pooled (intervention and control group) data, showed that participants who achieved ≥5% weight loss at year one had 64% lower T2D incidence (hazard ratio 0·36 (95% CI 0·22 to 0·56); P<0·001), those who maintained this at year two had 79% lower T2D risk (hazard ratio 0·21 (95% CI 0·09 to 0·49) P<0·001), and those who maintained this at year three had 89% lower T2D risk (hazard ratio 0·11 (95% CI 0·04 to 0·35) P<0·001) (Figure 4). There were no statistically significant differences in any of the baseline variables (as listed in Table 1) for those who met, or did not meet, these weight loss goals, other than a slightly lower baseline age for those achieving the weight loss goal in the first year (54·4 years compared with 56·0 years; P = 0·02). Analysis of effects of weight loss using only intervention group data produced similar results.

Cases achieving ≥5% weight loss at Year 1 numbered 194 (I = 144, C = 50). Results of Cox- regression survival analysis of T2DM incidence in the intervention compared with the control group showed HR 0.41(95% CI 0.16 to 1.03) p = 0.06 (B (SE) −0.90 (0.47)).

### Consideration of Risk Score and Hyperglycaemia Cut-point Values for Risk Identification

The number of EDIPS participants (all of whom had IGT) who would have been identified at baseline by FPG or HbA_1c_ cut points, as recommended by NICE, ADA and WHO, [2], [22], [23], [25] are shown in Table 2, with Cox-regression hazard ratios for progression to T2D. Applying the proposed NICE guidance FPG and HbA_1c_ cut-points to the EDIPS cohort of adults with IGT would have identified 66.8% (hazard ratio for progression to T2D 0·48 (95% CI 0·30 to 0·75) P<0.001) using FPG cut-points, but only 17.9% (hazard ratio 0·34 (95% CI 0·16 to 0·72) using HbA_1c_ cut-points, whereas applying the ADA cut points for HbA_1c_ would have identified 42.2% (hazard ratio 0.38 (95% CI 0.19 to 0.76) of EDIPS participants.

**Table 2:** EDIPS cases identified by high-risk FINDRISC^10^ score or by cut-points for fasting plasma glucose (FPG) and glycated Haemoglobin (HbA1c) as specified by NICE, ADA and WHO with risk statistics for intervention and control groups compared.

	Moderate or lower risk	High or greater risk	
**FNDRISC ^a 10^**	**<15**	**≥15**	
*EDIPS cases n (%)*	*387 (51·7))*	*330 (44·1)*	
*Hazard ratio (95% CI) P value and B(SE) for* *intervention/control incidence*	*0·62 (0·37 to 1·05)* *P = 0·072 −0.5(0.3)*	*0·30 (0·20 to 0·51)* *P<0·001 −1.2(0.3)*	
**FPG (mmoll** ^−**1**^ ** )**	**Lower risk**	**High risk**	**Diabetic range**
NICE ranges	**<5·5**	**5·5 to 6·9 (99 to 125** **mg/dl)**	**≥7·0**
*EDIPS cases n (%)*	*178 (23·8)*	*500 (66·8)*	*71 (9·5)*
*Hazard ratio (95% CI) P value and B(SE) for* *intervention/control incidence*	*0·29 (0·12 to 0·71)* *P = 0·007 −1.2(0.5)*	*0·48 (0·30 to 0·75)* *P<0·001 −0.7(0.2)*	
ADA ranges	**<5·6**	**5·6 to 6·9 (100 to 125 mg/dl)**	**≥7·0**
*EDIPS cases n (%)*	*214 (28·6)*	*464 (61·9)*	*71 (9·5)*
*Hazard ratio (95% CI) P value and B(SE)* *for intervention/control incidence*	*0·26 (0·11 to 0·61)* *P = 0·002 −1.4(0.4)*	*0·51(0·32 to 0·82)* *P = 0·005 −0.7(0.2)*	
WHO ranges	**<6·1**	**6·1 to 6·9 (110 to 125 mg/dl)**	**≥7·0**
*EDIPS cases n (%)*	*404 (53·9)*	*268 (35·8)*	*71 (9·5)*
*Hazard ratio (95% CI) P value and B(SE)* *for intervention/control incidence*	*0·43 (0·24 to 0·77)* *P = 0·005 −0.8(0.3)*	*0·40 (0·22 to 0·71)* *P = 0·002 −1.0(0.3)*	
**HbA1c^b^ (%)**	**Lower risk**	**High risk**	**Diabetic range**
NICE and UK-NSC ranges	**<6·0**	**6·0 to 6·4 (42–47 mmol/mol)**	**≥6·5**
*EDIPS cases n (%)*	*539 (72·0)*	*134 (17·9)*	*59 (7·9)*
*Hazard ratio (95% CI) P value and B(SE)* *for intervention/control incidence*	*0·44 (0·27 to 0·68)* *P<0·001 −0.8(0.2)*	*0·34 (0·16 to 0·72)* *P = 0·005 −1.1(0.4)*	
ADA ranges	**<5·7**	**5·7 to 6·4 (39–47 mmol/mol)**	**≥6·5**
*EDIPS cases n (%)*	*357 (47·7)*	*316 (42·2)*	*59 (7·9)*
*Hazard ratio (95% CI) P value and B(SE)* *for intervention/control incidence*	*0·42 (0·26 to 0·70)* *P<0·001–0.8(0.3)*	*0·38 (0·19 to 0·76)* *P = 0·006 −1.0(0.4)*	

The number of EDIPS cases that would have been identified at baseline by FINDRISC scores only, and combined with high-risk FPG and HbA1c cut-points (mimicking the NICE guidance proposed two stage screening process [2] ) are shown in Table 3. Applying the FINDRISC cut point for moderate risk (≥12) to the EDIPS IGT cohort would have identified 69·3% (hazard ratio 0·42 (95% CI 0·20 to 0·51) P<0·001) of participants. A combination of FINDRISC ≥12 and FPG 5·5 to 6·9 mmoll^−1^ would have identified 49·4% (hazard ratio 0·48 (0·28 to 0·83) P<0·001) whereas a combination of FINDRISC ≥12 and HbA1c 6·0 to 6·4% would have identified only 13·7% (hazard ratio 0·46 (95% CI 0·24 to 0·76) P = 0·08) of EDIPS participants [24].

**Table 3:** EDIPS cases identified by FINDRISC^10^ and high risk cut-points for fasting plasma glucose (FPG) and glycated Haemoglobin (HbA1c) as specified by NICE, ADA and WHO with risk statistics for intervention and control groups compared.

	FINDRISC score cut-points	
	Moderate or greater risk (≥12)	High or greater risk (≥15)
**FINDRISC** a **score only**		
*EDIPS cases n (%)*	*519 (69·3)*	*330 (44·1)*
*Hazard ratio (95% CI), P value and B(SE) for intervention/control incidence*	*0·43 (0·28 to 0.66)*	*0·30 (0·20 to 0·51)*
	*P<0·001–0.8(0.2)*	*P<0·001 −1.2(0.3)*
**FPG (mmoll** ^−**1**^ ** )**		
NICE high risk range (5·5 to 6·9)		
*EDIPS cases n (%)*	*354 (49·4)*	*224 (31·2)*
*Hazard ratio (95% CI), P value and B(SE) for intervention/control incidence*	*0·48 (0·28 to 0·83)*	*0·39 (0·20 to 0·78)*
	*P = 0·008 −0.9(0.4)*	*P = 0·008 −0.9(0.4)*
ADA high risk range (5·6 to 6·9)		
*EDIPS cases n (%)*	*328 (45·7)*	*209 (29·1)*
*Hazard ratio (95% CI), P value and B(SE) for intervention/control incidence*	*0·51 (0.30 to 0.87)*	*0·41 (0·21 to 0·83)*
	*P = 0·014 −0.7(0.3)*	*P = 0·007 −0.9(0.4)*
WHO high risk range (6·1 to 6·9)		
*EDIPS cases n (%)*	*198 (27·6)*	*62 (8·9)*
*Hazard ratio (95% CI), P value and B(SE) for intervention/control incidence*	*0·37 (0.23 to 0.64)*	*0·31 (0·13 to 0·73)*
	*P = 0·003 −1.0(0.3)*	*P = 0·007 −0.9(0.4)*
**HbA1c** b **(%)**		
NICE and UK-NSC high risk range (6·0 to 6·4)		
*EDIPS cases n (%)*	*96 (13·7)*	*62 (8·9)*
*Hazard ratio (95% CI) P value and B(SE) for intervention/control incidence*	*0·46 (0·19 to 1·10)*	*0·35 (0·12 to 1·00)*
	*P = 0·08 −0.8(0.4)*	*P = 0·05 −1.1(0.5)*
ADA high risk range		
*EDIPS cases n (%)*	*227 (32·4)*	*158 (22·6)*
*Hazard ratio (95% CI),P value and B(SE) for intervention/control incidence*	*0·43 (0·24 to 0·76)*	*0·28 (0·13 to 0·59)*
	*P = 0·004 −0.8(0.3)*	*P = 0·001 (−1.2(0.4)*

a n = 717.

b n = 732.

## Discussion

### Principal Findings

The analysis presented here reinforces existing evidence for the effectiveness of intensive lifestyle interventions to prevent T2D in adults with IGT and provides evidence for the generalisability of such interventions to European populations. Incidence of T2D was reduced by 57% in the intervention group compared with the control group. Explanatory analysis supported a ≥5% weight loss goal, as advocated in the EDIPS protocol and by NICE, [2] in this population and highlighted the importance of sustained weight loss long term (>1 year). Recently proposed cut-points, using a risk score (such as FINDRISC) and a blood test (FPG or HbA_1c_), to identify individuals at high-risk would have identified substantially different proportions of the EDIPS cohort of adults with IGT with little variation in the hazard ratios for T2D incidence. This analysis demonstrates the difficulties in efficiently identifying a population for targeting interventions, where progressive risk and evidence of potential to benefit, and intervention cost and capacity to deliver, are appropriately balanced.

### Strengths and Limitations of the Study

The EDIPS RCTs were conducted in three culturally different populations, recruited over a 10 year period, and the consistency of findings in different countries confirms the generalisability of the lifestyle intervention originally designed for the DPS. We might have expected the results of earlier diabetes prevention trials to influence control group participants in the later EDIPS cohorts, thus diluting the intervention effect, but there was little evidence that this occurred.

EDIPS was conducted in a population of white European ethnicity. In people of South Asian or African origin ethnicity, T2D risk is elevated. Trials elsewhere have demonstrated efficacy of lifestyle-based interventions for T2D prevention in these populations, [26] but not so far in Europe, although trials among South Asians living in Scotland, [27] London (G A Hitman personal communication), Leicester (K Khunti personal communication) and Oslo [28] are underway.

The EDIPS protocol was common to all three cohorts. The approach recognised the necessity for some local variation in intervention delivery to achieve cultural resonance, but the intervention goals, intensity and mode of delivery were similar. Changes to the diagnostic criteria for T2D since the design of EDIPS would have excluded some participants because they would have been diagnosed with T2D during recruitment. [22], [23] However, analysis of a sub-set of cases with FPG≥7·0 mmoll^−1^ and HbA1C≥6·5% removed did not alter the observed intervention effect. Diagnosis of T2D at annual follow-up was based on two hour venous plasma glucose values in an OGTT, [18] thus some people were retained in the study with FPG≥7·0 mmoll^−1^ or HbA1C≥6·5% who may have been diagnosed with T2D under later criteria [22], [23].

### Comparisons with Other Studies

The 57% overall reduction in T2D incidence, with a NNT of 7·4, observed in EDIPS is in line with outcomes of other diabetes prevention RCTs; meta-analysis of diet and/or exercise intervention studies has shown a pooled hazard ratio of 0·51 (95% CI 0·44 to 0·60) and NNT of 6·4. [6] The recently published DE-PLAN-CAT-PREDICE study [29] screened participants using FINDRISC (≥14) with subsequent OGTT to identify IFG or IGT (WHO classifications). [22], [23], [29] Comparison of the lifestyle intervention and standard care control groups showed risk reduction of 36·5%. This lower effectiveness may reflect the different study population (both different ethnicity and inclusion of IFG identified high risk participants) or different intervention, or both.

Recently data were published from a trial [30] where the primary inclusion criterion was IFG (ADA range: FPG 5·6 to 6·9 mmoll^−1^). [24] Overall, lifestyle intervention reduced T2D risk by 44%. However, post-hoc sub-group analyses showed a positive intervention effect in the IFG+IGT group (HR 0·41 (95% CI 0·24 to 0.69), and IFG+HbA_1c_ (HbA1c≥5.6%) group (HR 0·24 (95% CI 0·12 to 0·48) only. In people with isolated IFG, who comprised almost 60% of participants, no effect was observed (17% non-significant increase in risk).

Weight loss is attractive for monitoring compliance with interventions in routinely provided services, because it can be easily and objectively measured. [2] By pooling data from intervention and control groups for the analysis of weight loss and T2D incidence, we sought to evaluate the utility of sustained weight loss as an intermediate health outcome. Our explanatory analysis of the EDIPS data set of adults of white European ethnicity with IGT showed that achieving ≥5% weight loss at one year reduced T2D incidence by 64%, with enhanced effect if sustained at two or three years. Analysis of T2D incidence in those achieving ≥5% weight loss at Year 1 suggests that additional features of the intervention, such as increased physical activity and improved nutrition, contributed to the reduction in T2D incidence in these intervention group participants who also lost more weight on average and retained their weight loss for longer.

Translational studies to date, with the exception of the GOAL [31] and the DE-PLAN-CAT-PREDICE studies, [29] have had follow-up of one year or less and have focussed on weight loss as an intermediate health outcome. In a behaviour change RCT it is impossible to exclude self-help within the control group, especially where giving basic advice is ethically appropriate and regular monitoring is required for those at risk. In our qualitative evaluation, nested within the EDIPS-Newcastle trial, many participants reported that it took two years to establish lifestyle changes, suggesting that prolonged intervention may be required to maximise weight loss maintenance and thus effective prevention of T2D in those at high-risk [32].

The mean duration of the EDIPS trials was 3.1 years. Sustained reduction in the incidence of T2D and the mediating effect of weight loss have been shown in follow-up studies of the DPS [14] and the US Diabetes Prevention Programme. [13] However, in the Indian diabetes prevention study [33] a small weight loss in the lifestyle intervention group at six months was not sustained and the efficacy of weight loss for T2D prevention in people of South Asian ethnicity remains unclear. Whilst reducing progression to T2D in individuals at high-risk is important, there is also a need for population level interventions to reverse the rise in obesity in order to reduce T2D incidence [3].

### Possible Mechanisms and Implications for Clinicians and Policymakers

Translating evidence on the effectiveness of lifestyle intervention for prevention of T2D in adults with IGT into cost-effective, scalable interventions is challenging, especially in an era of budgetary constraints.

Among these challenges is the efficient identification of individuals at high-risk for effective, targeted intervention. Translational studies focus on risk identification using prospective risk scores. [34] For example, recent studies in Spain and Finland have demonstrated the effect of lifestyle intervention where recruitment was based on FINDRISC. [29], [35] The screening strategy proposed in the NICE guidance advocates a risk score (such as FINDRISC), followed by a single blood test (either FPG or HbA1c) to identify individuals at high T2D risk. [2] Applying the cut-points proposed by NICE to the EDIPS cohort shows that determining pragmatic, acceptable, low cost risk assessment with high predictive value in identifying individuals with the greatest potential to benefit from a T2D prevention intervention remains a challenge. The optimum combination of risk-score and blood test values for use in routine practice remains unclear and further research is needed, including economic evaluation.

Our explanatory analysis suggests that sustained weight loss at two and three years enhanced the intervention effect in the EDIPS cohort. The most efficient duration and intensity of lifestyle interventions to maintain weight loss [36] or to prevent T2D in individuals at high-risk remain uncertain. [2] The economic modelling undertaken for the NICE guidance development suggests that screening and intervention for T2D prevention is highly likely to be cost-effective, at a threshold of £10,000 per quality adjusted life year (QALY) gained or less, but the authors acknowledge that the results are based on assumptions about prevention effectiveness in individuals at high-risk identified by criteria other than IGT. [2] Our analyses provide an indication of intervention effectiveness, identified by a range of risk criteria.

### Unanswered Questions and Future Research

The screening strategy proposed in the NICE guidance is pragmatic, but our results show that the proposed cut-points for HbA_1c_ or FPG would identify different high-risk groups from those with IGT. Research is needed to demonstrate the effectiveness of lifestyle interventions on T2D incidence in populations identified by different combinations and ranges of risk scores, FPG and HbA_1c_ values to identify the optimum risk assessment strategy in terms of both patient benefit and cost-effectiveness. The incidence and prevalence of T2D are socio-economically patterned and there is therefore a need to develop lifestyle interventions for T2D prevention that are accessible, culturally adapted and which reduce social inequalities in outcomes. [37] However, the evidence to date, including the present study, suggests that lifestyle interventions are effective in diverse human populations.

Robust evaluations of pragmatic interventions for T2D prevention are needed that can be delivered efficiently in the context of routine health care provision and with sufficient duration for T2D incidence to be the primary outcome.

### Conclusion

The European Diabetes Prevention Study reinforces evidence that intensive lifestyle intervention can prevent T2D in adults with IGT, and provides evidence of wider generalisability in European populations. Analyses demonstrated the preventive effect of ≥5% weight loss, especially if maintained long term, which suggests the utility of a ≥5% weight loss target for intervention monitoring.

Proposed blood test cut-points for high–risk would have identified different proportions of the EDIPS cohort, with similar intervention benefit. Identification of high-risk individuals for provision of T2D prevention should consider risk profile along with potential to benefit, as well as overall cost-effectiveness and equity of outcomes.

## Supporting Information

Checklist S1 **CONSORT checklist.**
(DOC)

Protocol S1 **European Diabetes Prevention Study Protocol.**
(DOC)
