# Supplementary material for: Importance of Weight Loss Maintenance and Risk Prediction in the Prevention of Type 2 Diabetes: Analysis of European Diabetes Prevention Study RCT
Source: PLoS One. 2013 Feb 25;8(2):e57143. doi: 10.1371/journal.pone.0057143 (PMC3581561; doi:10.1371/journal.pone.0057143)
Supplement: Protocol S1 — European Diabetes Prevention Study Protocol. (DOC) [file pone.0057143.s002.doc]

# Protocol S1

PREVENTION OF DIABETES IN PERSONS WITH IMPAIRED

GLUCOSE TOLERANCE

A CLINICAL TRIAL USING PHYSICAL ACTIVITY AND DIETARY MODIFICATION

SHORT TITLE: DIABETES PREVENTION STUDY (DPS)

**PROTOCOL AND MANUAL OF OPERATIONS**

May 1996

Jaakko Tuomilehto, Matti Uusitupa and co-workers

CONTENTS

Page

Table of contents ii

Detailed table of contents iii

Abbreviations vii

Chapter

1 SUMMARY 1

2 STUDY RATIONALE AND OBJECTIVES 2

3 STUDY DESIGN 23

4 VISIT AND DATA SCHEDULE 30

5 ELIGIBILITY, SCREENING, AND EXAMINATION PROCEDURES 32

6 TREATMENT AND TESTING PROCEDURES 39

7 ENDPOINTS CLASSIFICATION 44

8 DATA ANALYSIS 47

Appendix

A GLOSSARY 50

B CENTERS AND INVESTIGATORS 51

C BIBLIOGRAPHIC REFERENCES 52

DETAILED TABLE OF CONTENTS

Table of contents ii

Detailed table of contents iii

Abbreviations vii

CHAPTER 1. SUMMARY 1

CHAPTER 2. STUDY RATIONALE AND OBJECTIVES 2

2.1 INTRODUCTION 2

2.2 INCIDENCE OF DIABETES 2

2.2.1 Stages in development of diabetes and its complications.

2.2.2 Diagnosis of IGT and prediction of diabetes.

2.2.3 Previous intervention studies in IGT.

2.3 JUSTIFICATION FOR THE PRESENT STUDY 8

2.3.1 Justification of the study design.

2.3.2 Justification for performance in Scandinavia.

CHAPTER 3. STUDY DESIGN 23

3.1 STUDY PURPOSE AND DESIGN SUMMARY 23

3.1.1 Purposes.

3.1.2 Primary Endpoint.

3.1.3. Secondary endpoints.

3.1.4 Subjects.

3.2 SUBJECT ELIGIBILITY AND EXCLUSION CRITERIA 24

3.2.1 Definitions of diabetes and IGT.

3.2.2 Eligibility and exclusion criteria.

3.3 SCREENING FOR IGT 25

3.4 ELIGIBILITY CRITERIA 25

3.5 REGISTRATION, RANDOMIZATION, AND STRATIFICATION 26

3.5.1 Treatment assignment and registration.

3.5.2 Randomization to treatment.

3.5.3 Stratification.

3.5.4 Registration.

3.6 FOLLOW-UP 26

3.7 MASKING 27

3.7.1 Diet-exercise intervention.

3.8 SAMPLE SIZE AND ENROLLMENT GOAL 27

3.9 PRETEST OF PROCEDURES AND DATA FORMS 29

CHAPTER 4. VISIT AND DATA SCHEDULE 30

4.1 OVERALL SCHEDULE 30

4.2 SCHEDULED VISITS 30

4.2.1 Screening Visits.

4.2.2 Enrollment Visit.

4.2.3 Interim Visits.

4.2.4 Annual Visits.

4.3 TIME WINDOWS 31

CHAPTER 5. ELIGIBILITY, SCREENING, AND EXAMINATION PROCEDURES 32

5.1 ELIGIBILITY AND EXCLUSION CRITERIA 32

5.1.1 Eligibility criteria

5.1.2 Exclusion criteria

5.2 SCREENING FOR IGT 34

5.2.1 Population selected for screening.

5.2.2 Conditions for the screening examination.

5.2.3 The oral glucose tolerance test.

5.3 SCREENING EXAMINATIONS 35

5.3.1 Conditions for examination

5.3.2 Content of first screening examination.

5.3.3 Content of second screening examination

5.4 ANNUAL EXAMINATIONS 37

5.4.1 Conditions for examination.

5.4.2 Content of annual examinations.

5.5 POST-TREATMENT FOLLOW-UP 37

CHAPTER 6. TREATMENT AND TESTING PROCEDURES 39

6.1 DIET-EXERCISE TREATMENT 39

6.1.1 Standard diet-exercise treatment group.

6.1.2 Special diet-exercise treatment group.

6.2 MONITORING FOR COMPLIANCE AND ENDPOINTS 40

6.2.1 Interim visits.

6.2.2 Annual examination and OGTT.

6.2.3 Diet-exercise treatment.

6.2.4 Physical activity assessment

6.2.5 Assessment of diet

CHAPTER 7. END-POINTS CLASSIFICATION 44

7.1 WORSENING FROM IGT TO DIABETES 44

7.2 CARDIOVASCULAR END POINTS 44

7.2.1 Definite myocardial infarction.

7.2.2 Possible myocardial infarction.

7.2.3 Ischemic cardiac arrest.

7.2.4 Sudden cardiac death.

7.2.5 Unwitnessed cardiac death.

7.2.6 Angina.

7.2.7 Intermittent claudication.

7.2.8 Stroke.

7.2.9 Atherosclerosis

7.3 END-POINT CLASSIFICATION 46

CHAPTER 8. DATA ANALYSIS 47

8.1 INTRODUCTION 47

8.2 PRETREATMENT CHARACTERISTICS 47

8.3 OUTCOME VARIABLES 48

8.3.1 Events.

8.3.2 Continuous variables.

8.4 CLINICAL COURSE AND PROGNOSIS 49

APPENDIX A. GLOSSARY 50

APPENDIX B. CENTERS AND INVESTIGATORS 51

APPENDIX C. REFERENCES 52

ABBREVIATIONS

Coronary Heart Disease

Cardiovascular Disease

IGT Impaired Glucose Tolerance

DPS Diabetes Prevention Study. Short version of Prevention of Diabetes in Persons with Impaired Glucose Tolerance: A Clinical Trial.

HbA1c Glycosylated hemoglobin, fraction A1c

HPLC High pressure liquid chromatography

NIH National Institutes of Health

OGTT Oral glucose tolerance test

WHO World Health Organization

Insulin-dependent diabetes

Non-insulin-dependent diabetes

CHAPTER 1. SUMMARY

A randomized clinical trial will be performed in middle-aged Finnish people to determine whether treatment of obese, sedentary adults with impaired glucose tolerance (IGT) will prevent or delay the development of type 2 diabetes.  The study will be conducted in several centers, will include 560 subjects, and will have a mean duration of 5.5 years per subject.  Subjects will be allocated at random to a standard or special diet-exercise treatment. Subjects will be enrolled over a two-year time period. Those enrolled during the first year will be treated for 6 years, and those enrolled during the second year will be treated for 5 years, so that all treatment cycles will finish during the final year of the study. The study is designed to have a power of 80% to detect a reduction of 30% or more in the incidence of diabetes by the diet-exercise program.

The applicant investigators have already demonstrated the feasibility of screening for IGT in their countries and providing effective interventions of the type to be used in this clinical trial. **This trial belongs to the category of "interventions in IGT and early diabetes in high-risk non-insulin-dependent diabetes mellitus populations on development of diabetes and its complications", which was recommended by the U.S. National Diabetes Advisory Board (1991) as a priority area for research in diabetes. No such studies have been initiated in this important issue so far, in spite of these unanimous recommendations by the world experts.**

CHAPTER 2. STUDY RATIONALE AND OBJECTIVES

2.1 INTRODUCTION

IGT is a condition defined by an abnormal response to an oral glucose tolerance test. Although persons with IGT do not show any manifes­tations of diabetes and are not considered to have diabetes (which can be diagnosed by a more extreme abnormality of glucose tolerance), IGT is clinically important because persons with IGT are at increased risk of developing diabetes and coronary heart disease. In 1980 the World Health Organization (WHO) adopted a classification of diabetes and other categories of glucose tolerance, which was revised in 1985 (World Health Organization, 1985). The two common types of diabetes are type 1, or insulin‑dependent diabetes, and type 2, or non-insulin‑dependent diabetes.  Diabetes can also be secondary to other diseases.

In addition to a new classification, new diagnostic criteria for diabetes and IGT have been widely adopted.  The diagnosis of diabetes may be established on the basis of casual plasma glucose concentra­tions ≥11.1 mM (200 mg/dl) or the presence of fasting venous plasma glucose concentrations ≥7.8 mM (140 mg/dl) on more than one occasion, or by oral glucose tolerance tests meeting the criteria shown in Table 3.1.  IGT is diagnosed when the criteria for diabetes are not met, but the 2 hour post‑load plasma glucose is from 7.8 to 11.0 mM (140 to 199 mg/dl).

2.2 INCIDENCE OF DIABETES

Diabetes mellitus is an increasingly common disease with a large impact on society and a long, complicated, and poorly understood natural history.  These factors make the question of the possibility of preventing diabetes both important and difficult to resolve.  Diabetes is characterized in the untreat­ed state by chronic hypergly­cemia.  In its most overt form there are classical symptoms such as poly­dipsia, polyuria, polyphagia and weight loss which are directly attributable to hyperglycemia, but these are often not present.  The syndrome is also characterized by the development of microvascular complica­tions, such as retinopathy and nephropathy, neuropathy, and macrovascular complications which give rise to excessive rates of coronary heart disease and peripheral vascular disease. These complications account for the majority of morbidity and mortality associated with diabetes.  Diabetes seriously affects the quality of life because of these complications, and life is shortened, with diabetes ranking seventh as a cause of death in the U.S. in 1980. A recent study in Finland showed that diabetes increased the risk of death in 65-74 year old men fivefold (Stengård et al 1992).

2.2.1 Stages in development of diabetes and its complications. IGT is a critical stage in the development of diabetes, because it is readily detectable and treatment may prevent or delay its progression.  In general a number of stages relating to the course and development of diabetes may be defined which apply to both common types of the disease.  **The first stage is genetic susceptibility**.  At present this cannot be specifically identified in the general population. **Second is the state of potential abnormality of glucose tolerance**, a statistical risk class including subjects who have normal glucose tolerance but who by virtue of other charac­teristics have a substan­tially increased risk for the development of diabetes.  **Third is IGT,** a phase in the development of type 2 diabetes, and, as only recently recognized, in type 1 also.  **Fourth is diabetes without complica­tions**, a stage in which chronic hyperglycemia is present and symptoms attribu­table to hyperglycemia may or may not be present.  **Fifth is diabetes with vascular complications,** but without associated symptomatology or disability.  **Last is diabetes with disability**, when complications of diabetes lead to functional impairment.  Progression through these stages is not inevitable.  For example many people with characteristics which are risk factors for abnormal glucose tolerance never progress to diabetes, and many people with IGT remain nondiabetic after many years of follow-up.  Nonetheless, many people do progress, and for this reason it is imperative to determine if this progression can be halted.  Potentially, intervention at any of stage may prevent progression to a later stage.  IGT is the earliest easily recog­nizable stage in this process, and there is some evidence that its progression to diabetes can be prevented.  Hence efforts to prevent diabetes and its complications should be directed towards those with IGT.

This conclusion is in agreement with a recent recommendation of the U.S. National Diabetes Advisory Board that "interventions in IGT and early diabetes in high-risk non-insulin-dependent diabetes mellitus populations on development of diabetes and its complications" should be one of the top two priorities for clinical trials (National Diabetes Advisory Board, 1991).

2.2.2 Diagnosis of IGT and prediction of diabetes. IGT is quantitatively a major problem.  The prevalence increases with age, and, by WHO criteria, reaches 23% at ages 65‑74 years in the U.S. population according to the National Health and Nutritional Examination Survey (Harris, et al, 1987). Several population studies have shown people with IGT to be at increased risk of developing diabetes, although if their glucose tolerance does not deteriorate, they are not at risk of diabetic complications.  There is, however, evidence that they are at increased risk of coronary disease (Fuller, et al, 1980).  There have been at least eleven studies of the rate at which persons with IGT progress to overt diabetes. These studies, summarized in Table 2.1, are difficult to compare because different diagnostic criteria for IGT and diabetes have been used, differing especially for IGT. Most of these predated the WHO criteria (World Health Organization, 1985) for glucose tolerance and used criteria which are much less stringent, i.e. they included many subjects who would be normal by WHO criteria. Thus in those studies using WHO criteria, such as the studies in Malmöhus County (in a subset of subjects meeting WHO criteria for IGT), Nauru, Malmö, Malta, San Antonio, and the Pima Indian study, the incidence rates of diabetes were higher, at 19-61% in 5-10 years. In the others, the two-hour blood glucose at entry was consistently a strong predictor of future diabetes, so it is reasonable to estimate that had the more stringent WHO criteria been used, the incidence rates might have been higher and similar to those in the studies in which WHO criteria were used. The best estimates of incidence rates to be anticipated in subjects with IGT by WHO criteria are based on those studies shown in Table 2.1 in which WHO criteria were used.

As the incidence rates are in general higher in non-Causasian populations, the incidence has been estimated in Caucasians from the three studies in middle-aged Caucasians using WHO criteria: Malmöhus, Malmö, and San Antonio. If the 5-year cumulative incidence rates in these three studies are averaged, weighted by sample size (excluding the treated subjects in Malmö), the estimated 5-year incidence rate for Caucasians is 16%. For our study this is an underestimate because of the additonal entry criteria of obesity, which is associated with higher incidence of diabetes in persons with IGT at baseline (Saad, et al, 1988; Haffner, et al, 1990), and because some of the subjects will be followed for six, rather than five, years (depending on time of enrollment).

Most of these estimates are based on follow-up of persons with IGT diagnosed by one OGTT without corroborative evidence. Some corroborative evidence would be highly desirable before udertaking a long-term intervention in IGT. One possibility is to repeat the OGTT after a short interval of time and enroll only those persons meeting WHO criteria for IGT on both occasions. Another possibility would be to use HbA1c as an entry criteria. Among Pima Indians with IGT, those in the top 30% of the distribution of HbA1c had 2.47 times the incidence of diabetes during follow-up as did those in the lower 70%, or 1.74 times the incidence as in all the subjects with IGT combined (Little et al, submitted).

Although we are unaware of estimates of the multiplier of 1.74 for the incidence of diabetes in IGT diagnosed with repeated OGTTs, we will use this number to estimate the 5-year incidence of diabetes to be 1.74 times 16% = 28% in persons to be enrolled in the clinical trial.

2.2.3 Previous intervention studies in IGT. There have been few attempts to determine the effect of intervention on IGT.  Clinical research studies have shown that the administration of a hypocaloric diet to obese subjects with IGT leads to an improvement in glucose tolerance and, possibly, insulin action.  Similarly, sulphonyl­ureas can both increase insulin secretion and reduce insulin resistance in subjects with IGT or type 2 diabetes, and biguanide drugs reduce hyperglycemia by improving peripheral insulin action and inhibiting intestinal glucose absorption (see below, section 2.3.1).  There have been only a few attempts to examine the effects of diet on the incidence of diabetes in population samples.

England. In the Bedford (Keen, et al, 1982) and Whitehall (Jarrett, et al, 1979) studies in England, there was no discernable effect of either diet or oral antidiabetic agents on the incidence of subsequent diabetes.  In Bedford the rates of decompensation were very similar in those randomly assigned to tolbutamide or placebo. Similarly in the Whitehall study, assignment to treatment with phenformin had no benefit.  Subjects were given either phenformin or a placebo, and either advised to limit sucrose intake (with no other dietary advice) or prescribed a limited carbohydrate diet. The incidence of diabetes did not differ significantly by these treatment groups.

Malmöhus, Sweden. In a third study, the Malmöhus study in Sweden (Sartor, et al, 1980), adult subjects with IGT were randomly assigned to one of three groups, all of which received diet treatment.  Patients in two of the groups also received tablets, either tolbutamide or a placebo.  In ten years, the incidence of overt diabetes was 12% (10% in those assigned to tolbutamide and 13% in the others). While the incidence of diabetes was lower in those assigned to tolbutamide, the differences between the treatment groups was not statistically significant. These data were also analyzed according to estimates of compliance in tablet‑taking.  These estimates were based both on history and, toward the end of the study, on measurement of serum tolbutamide concentra­tions.  Of the 23 subjects who continued to take tolbutamide, none developed diabetes.  This is a small number of subjects, and their incidence rate is not statist­ically signif­icantly different from the 15% in all other subjects given diet treatment, with or without drug or placebo assignment.  Furthermore, these subjects represented less than half of those randomized to this group.  It is unknown whether there was a true therapeutic effect or whether those who elected to continue tolbutamide were a self‑selected group who for other reasons had a lower incidence of diabetes.

Long-term follow-up of this trial was, however, more promising. Although the study-assigned treatments were carried out for 10 years, mortality follow-up was conducted for an additional 12 years. At 22 years after study entry, the incidence rate of fatal myocardial infarction in the men assigned to tolbutamide was only 0.26 times (95% confidence interval = 0.08 to 0.86) that in those not treated with tolbutamide, and the rate ratio for deaths from all causes was 0.59 (95% confidence interval 0.35 to 1.01) (Knowler, et al, in preparation). This suggests that whether or not the incidence of diabetes was reduced in this clinical trial, the treatment assignment had beneficial effects on cardiac and total mortality rates.

Malmö, Sweden. Eriksson and Lindgärde (1991) have recently completed a study of the feasibility of diet and exercise treatment of men with IGT in Malmö, Sweden. On the basis of population screening of 47-49 year old men, 217 subjects with IGT were identified. The criteria for IGT were similar to those proposed in the present study: the subjects had fasting blood glucose < 6.7 mM and two-hour blood glucose between 7.0 and 11.0 mM on two tests on two different days. The subjects were divided into two groups, 161 were treated with diet and exercise and 56 were a reference group. The groups were not assigned at random. The reference group were men referred to other clinics for specialized care, such as for hypertension or hyperlipidemia, and the remainder were treated by the investigators. The reference group were treated for their other medical conditions according to standard practice, but were not given specific treatment for hyperglycemia. The treated group were given dietary advice to reduce sugar and fat, increase complex carbohydrate and fiber, and lose weight if they were overweight. They were also trained in physical exercise by means of group exercise sessions for one hour, twice per week for the initial 6-12 months. Thereafter they were encouraged to continue exercising at home or in sports clubs. They were given no drug treatment for hyperglycemia, although some took drugs for other indications, e.g. hypertension. The treated subjects, but not the reference group, had a significant weight loss, most of which was maintained for 5 years. By the end of the 5-year treatment period, 11% of the treated subjects and 21% of the reference group had developed diabetes according to WHO criteria. Thus the incidence in the treated group was 0.5 times (95% confidence interval=0.3 to 1.0) that of the reference group. **This study is important in demonstrating the feasibility of carrying out a diet-exercise program for 5 years, and it suggests that the incidence of diabetes can be reduced by 50% by such treatment. A treatment effect was not definitely established, however, because the treatments were not assigned at random.**

Thus the question of preventing diabetes with diet or exercise is not settled, and there is an urgent need to replicate these studies.  If treatment of IGT with diet, or exercise will indeed delay or prevent diabetes, the detection and treatment of persons at this stage could drastically reduce the incidence of the serious complications and excess mortality of type 2 diabetes.

2.3 JUSTIFICATION FOR THE PRESENT STUDY

2.3.1 Justification of the study design. Because the question of whether diabetes can be prevented in subjects with IGT is so important but not yet clearly answered, we propose a new clinical trial to test the hypothesis.  The efficacy of diet-exercise program will be tested. There are several reasons for suspecting these treatments may be effective.  Among those with IGT, obesity, the degree of hyperglycemia, insulin resistance, and impaired insulin secretion have been identified as major risk factors for type 2 diabetes.  Thus therapeutic measures designed to reduce body weight and hyperglycemia might be effective in preventing the deteriora­tion of IGT to diabetes. This study will test a diet-exercise treatment program including exercise and a diet designed to imporove hyperglycemia and reduce body weight.

Diabetes and insulin resistance are now commonly agreed to increase the risk of CHD and stroke ( Pyörälä et al. 1983, Black 1990) over and above the other major cardiovascular risk factors. It is not known whether this excess risk could be reduced by preventing the worsening of glugose intolerance. Recently, developments in high-resolution B-mode ultrasound has made it possible to quantify mild and moderate asymptomatic lesion formation and also to monitor serial arterial change ( Pignoli et al 1986, Bond et al 1991, Riley et al 1991). Preliminary population based data from the large ARIC study in the US (the ARIC Investigators 1989) show that diabetic sujects had significantly larger carotic artery wall thicness even when stratifying for smoking, hypertension and hypercholesterolemia (Chambless et al 1992, unpublished data). As the secondary hypothesis our study will test whether the prevention of worsening of glucose intolerance will prevent atherosclerosis and/or acute CVD events.

Physical activity

Although the pathogenesis of non-insulin-dependent diabetes mellitus (NIDDM) is not fully understood, it is clear that at least three factors are important: a genetic predisposition to the disease; a defect in pancreatic β-cell function and a decrease in the action of insulin in insulin-sensitive tissues, including adipose tissue, skeletal muscle, and the liver; and a defect in β-cell pancreatic function (De Fronzo 1988). Conditions associated with the development of insulin resistance increase the risk of NIDDM greatly. Chief among these are obesity and advancing age. More recently, it has been recognized that hypertension may be associated with resistancee to insulin and hyperinsulinemia (Modan et al 1985). So, too, may be the increase in plasma of very-low-density lipoprotein and the decrease in high-density-lipoprotein cholesterol that are characteristic of patients with NIDDM. Thus, insulin resistance and hyperinsulinemia may have a critical role in the pathogenesis of the constellation of metabolic abnormalities that occur frequently in patients with NIDDM - namely, obesity, hyperlipidemia, and hypertension. Conversely, interventions that prevent or reverse insulin resistance through alterations in life-style, such as increased physical activity and the prevention of obesity, may have a substantial protective effect.

Approximately 20 years ago, Björntorp and his colleagues suggested that physical training is associated with lower plasma insulin concentrations and improved insulin sensitivity (Björntorp et al 1972). They found that physically active middle-aged men who regularly participated in sports had much lower plasma insulin concentrations both while fasting and after the administration of glucose than did inactive men matched for age and weight who had similar blood glucose concentrations.

It was not determined in this cross‑sectional study whether the differences in insulin secretion and action were results of physical training or of other differences between physically active and sedentary persons. Further studies demonstrated that plasma insulin concentrations decreased in obese, insulin‑resistant subjects who participated in physical‑training programs, even when they had no change in body composition or oral‑glucose tolerance (Björntorp et al 1973). Subsequently, numerous investigators have demonstrated that both regular physical training and the actual activity of exercise increase insulin sensitivity, whereas deconditioning and physical inactivity are both associated with the development of insulin resistance (Horton 1986). The effects of physical training on insulin sensitivity are rather short‑ lived, however, and they mostly disappear within a few days of the discontinuation of regular exercise (Burstein et al 1985). Marked reductions were detected in a group of Australia Aboriginal subjects who reverted from living a modern to a traditional life‑style which involved changes in physical activity, as well as in diet (O'Dea 1984). In the Swedish study (Eriksson and Lindgarde 1991) 10‑14 % increase was observed in oxygen uptake for both diabetic and IGT groups who participated in the program over their baseline values. This was in contrast to the control groups in which a reduction of 5‑9 % was observed. Body weight increased by 0.5‑1.7 % in the control groups, whereas it was reduced by 2.3.‑3.7 % in the active intervention groups. Glucose tolerance became normal in more than 50 % of the intervention group, with the cumulative incidence of diabetes being 10.6 % in the IGT group after 6 years. Blood pressure, lipids, and hyperinsulinemia were also reduced. Mortality in the intervention groups was 33 % lower than that in the control group.

The mechanisms by which exercise and physical training increase insulin sensitivity are not yet fully understood. After a single session of exercise, glucose uptake by skeletal muscle is enhanced for several hours. This occurs without any increase in the binding of insulin to its receptor (Horton 1986). However, the number and activity of glucose-transporter proteins, particularly the Glut 4 isoform, are increased in plasma membranes of skeletal muscle after exercise (Goodyear et al 1990). In addition glycogen synthase activity is increased, resulting in increased synthesis of glycogen and increased nonoxidative disposal of glucose.

During training, the binding of insulin to its receptor is slightly increased in adipose tissue but not in muscle. However, both tissues have a substantial increase in total number of glucose transporters and enhanced glucose transport in response to insulin stimulation (Hirshman et al 1989). It now appears that a major effect of physical training is to increase the capacity for glucose transport into muscle and adipose tissue in response to insulin stimulation. Other factors, such as increased capillary density in muscle and increased intracellular glucose metabolism, may also be important in the adaptation to training.

Regular physical exercise has long been considered an important part of the treatment of NIDDM and is frequently prescribed, along with diet, oral hypoglycemic agents, or insulin. In addition to lowering blood glucose levels in the short term, exercise may improve long-term glucose control, as indicated by glycosylated hemoglobin concentrations. Insulin sensitivity is also increased. Other benefits of exercise extend to several cardiovascular risk factors, such as improvement in mild-to moderate hypertension, improved lipid profiles, and more effective weight reduction (Horton 1988, Eriksson and Lindgarde 1991).

Although a role for physical conditioning in the prevention of NIDDM has been suspected on the basis of population and cross-sectional studies, no effect of exercise independent of other factors has been identified until recently. In Malta, where the prevalence of NIDDM is one of the highest in Europe, a 2-year follow-up study as performed in a random sample of the population. 388 subjects were classified into three categories of physical activity and three categories of glucose tolerance. At baseline, physical activity was inversely associated with the 2-h post challenge blood glucose, and in multivariate analysis age, family history of diabetes and physical inactivity were the strongest predictors of 2h blood glucose (Schranz et al 1990). The age standardized risk of glucose intolerance, i.e. IGT or NIDDM was consistently and inversely related to the level of physical activity. Among subjects with normal glucose tolerance at baseline those with low physical activity had a 2.7 times higher risk of glucose intolerance during follow-up than those with high physical activity, and a 3.7-fold risk of glucose intolerance when both the subjects with normal and IGT at baseline were considered together. The results suggested that the protective effect of physical activity was independent of obesity, family history of diabetes and gender. Helmrich et al. (1991) present convincing evidence from a long-term study of the development of chronic disease in former college students (the University of Pennsylvania Alumni Health Study) that there is an inverse relation between energy expenditure in leisure-time physical activity and the development of NIDDM during the subsequent 15 years.

The Nurse's Health Study in the U.S. involved 87253 women aged 34-59 years free of diabetes at baseline (Manson et al. 1991). During 8 years of follow-up 1303 cases of NIDDM were confirmed. Women who engaged in vigorous exercise at least once per week had an age-adjusted relative risk (RR) of NIDDM of 0.67 (*p* < 0.0001) compared with women who did not exercise weekly. After adjustment for body-mass index, the reduction in risk was attenuated but remained statistically significant (RR = 0.84, *p* = 0.005). When analysis was restricted to the first 2 years after ascertainment of physical activity level and to symptomatic NIDDM as the outcome, age-adjusted RR of those who exercised was 0.5, and age and body-mass index adjusted RR was 0.69. Among women who exercised at least once per week, there was no clear dose-response gradient according to frequency of exercise. Family history of diabetes did not modify the effect of exercise, and risk reduction with exercise was evident among both obese and nonobese women. Multivariate adjustments for age, body-mass index, family history of diabetes, and other variables did not alter the reduced risk found with exercise.

Several key findings emerge from this prospective study of men who responded to detailed health questionnaires in 1962 and again in 1976. During this period, 3.4 percent of the men reported that diabetes mellitus had developed, with the age at the time of diagnosis ranging from 45 to 68 years. Significant risk factors included a parental history of diabetes, older age, a history of hypertension, the presence of obesity as measured by the body-mass index, and low levels of leisure-time physical activity. The protective effect of physical activity was independent of the other risk factors and was particularly strong among men who participated in vigorous sports, although participation in less vigorous activities was also protective. The occurrence of NIDDM decreased by 6 percent for each increment of 500 kcal per week expended in physical activity in the whole study group. Two thirds of the newly diagnosed cases of diabetes occurred among men who had the high-risk characteristics of obesity, hypertension, and a parental history of diabetes. In this subgroup, habitual physical activity had the greatest protective effect, decreasing the incidence of diabetes by 24 percent from the highest activity group to the lowest. These findings strongly support the position that persons who are at substantial risk for NIDDM should be encouraged to maintain a high level of physical activity in their daily lives.

The association between regular exercise and the subsequent development of NIDDM was examined in a prospective cohort study including 5 years of follow-up (Manson et al 1992). 21 271 US male physicians participated in the Physicians' Health Study, aged 40 to 84 years and free diagnosed diabetes mellitus, myocardial infarction, cerebrovascular disease, and cancer at baseline. At baseline, information was obtained about frequency of vigorous exercise and other risk indicators. During 105 141 person-years of follow-up, 285 new cases of NIDDM were reported. The age-adjusted incidence of NIDDM ranged from 369 cases per 100 000 person-years in men who engaged in vigorous exercise less than once weekly to 214 cases per 100 00 person-years in those exercising at least five times per week (P, trend_1_ <.001). Men who exercised at least once per week had an age-adjusted relative risk (RR) of NIDDM of 0.64 (95 % Cl, 0.51 to 0.82; P=.0003) compared with those who exercised less frequently. The age-adjusted RR of NIDDM decreased with increasing frequency of exercise. 0.77 for once weekly, 0.62 for two to four times per week, and 0.58 for five or more times per week (P, trend .0002). A significant reduction in risk of NIDDM persisted after adjustment for both age and body-mass index: RR, 0.71 (95 % Cl, 0.56 to 0.91;P=.006) for at least once per week compared with less than once weekly, and P_1_ trend_1_.009, for increasing frequency of exercise. Further control for smoking, hypertension, and other coronary risk factors did not materially alter these associations. The inverse relation of exercise to risk of NIDDM was particularly pronounced among overweight men.

Although the mechanism of the protective effect of regular exercise is not addressed in these few prospective studies, the large body of literature demonstrating that physical training is associated with lower plasma insulin concentrations and increased sensitivity to insulin in skeletal muscle and adipose tissue strongly suggests that this may be a critical factor. The other critical factor in preventing NIDDM is, of course, the prevention or treatment of obesity through dietary restriction and increased energy expenditure. It can also be expected that increased physical exercise and the prevention of obesity will substantially lower the incidence of hypertension and dyslipidemia, particularly in persons with insulin resistance or hypersinsulinemia as well as in those with NIDDM. Regular physical activity is an important component of a healthy life-style for all of us, but it is particularly important for those at increased risk for chronic diseases such as NIDDM, hypertension and hyperlipidemia (Horton 1991).

Studies on past history of physica activity, even those which have used the physical activity indicators for the past have shown that its effect on metabolic parameters migh persist for a long time (Frish et al 1986). Therefore, it will be important to have reliable data on physical activity at younger ages, since it may modify the outcome, i.e. the development of diabetes (King and Kriska 1991).

Obesity and fat distribution

Obesity has been implicated as a risk factor for NIDDM in cross-sectional (King et al 1984, King et al 1986, McLarty et al 1989) and longitudinal (Medalie et al 1975, Stanhope et al 1980, Knowler et al 1981, Keen et al 1982, Butler et al 1982, Balkau et al 1985, Haffner et al 1986, Ohlson et al 1988, Schranz 1989, Dowse et al 1991) studies. That BMI seems to be associated with increased risk of NIDDM in both sexes and all ethnic groups.

Centralized ditribution of body fat (variously measured and referred to as abdominal, upper body, truncal, or central obesity) has been implicated as a risk factor for NIDDM in Europeans (Butler et al 1982, Ohlson et al 1988, Ohlson et al 1985, Tuomilehto et al 1990), Mexican Americans (Haffner et al 1986, Haffner et al 1990), Native Americans (Szathmary et al 1983), and in both cross-sectional (Haffner et al 1986) and longitudinal (Ohlson et al 1988, Ohlson et al 1985, Haffner et al 1990) studies. A recent study in Mauritius found positive associations in four ethnic groups - both Hindu and Muslim Asian Indians, Chinese, and Croeles of predominatly African origin Dowse et al 1991). This study also found an association between risk of IGT and abdominal obesity, which has not been previously reported, indicating that fat distribution is important in determining risk of lesser degrees of glucose intolerance (Dowse et al 1991).

Haffner et al (1986) in cross-sectional data found that a centrality index (ratio of subscapular to triceps skin fold) predicted risk of NIDDM, independent of BMI, in Mexican-American and non-Hispanic white women but not in men. **These findings were confirmed in a Finnish cross-sectional study (Tuomilehto et al 1990).** Conversely, a longitudinal study in Swedish men found that WHR acted independently of BMI in conveying risk of NIDDM (Ohlson et al 1988). In Mauritius, waist to hip ratio (WHR) and BMI seemed to act independently for risk of both IGT and NIDDM in both sexes (Dowse et al 1991). Taken together, the data from the United States, Mauritius, Finnish and Swedish indicate that both overall body mass (as reflected by BMI) and the distribution of fat are important independent predictors of risk for glucose intolerance in both sexes, regardless of ethnic origin.

The mechanisms through which abdominal obesity and overall body mass may separately influence the risk of glucose intolerance remain speculative. There is evidence that insulin resistance may be primarily modulated by abdominal obesity, whereas insulin secretion (and tendency to hyperinsulinemia ) may be predominately related to overall body mass (Evans et al 1984, Peiris et al 1987, Björntorp et al 1988). After controlling for glucose and insulin levels, Saad et al (1988) found that obesity was not an independent predictor of progression from IGT to NIDDM in Pima Indians. Similarly, neither BMI nor centrality index reminaed significatn predictor of incident NIDDM in Mexican-American subjects after controlling for glucose and insulin (Haffner et al 1990). These findings may indicate that obesity and adverse fat distribution both act through insulin resistance in determining risk of NIDDM. However, body mass was an independent predictive factor, after controlling for insulin and glucose, in similar studies in Europids (Keen et al 1982), Japanese (Kadowaki et al 1984), and Nauruans (Sicree et al 1987), so the possibility that obesity may act through other pathway should not be discounted.

The extent to which obesity may be modifiable is unclear in the absence of information from population-based intervention programs. Weight loss is notoriously difficult in obese individuals (National Institutes of Health 1987), and the extent to which abdominal distribution of body fat is reversible is unclear. However, early evidence indicates that weight loss in individuals with abdominal obesity leads to improvements in WHR (Shimokata et al 1989), and have suggested that the metabolic abnormalities associated with abdominal obesity may be uniquely improved by physical training (Krotkiewski et al 1986).

**Even though primary prevention of diabetes was already proposed about 70 years ago (Joslin 1921), and more recently stressed by the WHO (1985) and the US National Diabetes Advisory Board (1991), there have been no previous properly designed studies to assess the value of a program aiming at reducing weight increasing physical activity and modifying of fat/carbohydrate intake in the diet in primary prevention of diabetes (Tuomilehto and Wolf 1987, Tuomilehto 1989).**

Dietary fats and carbohydrates

Although there is general agreement that diet should serve as the cornerstone in the treatment of individuals with diabetes, the exact nature of the diet remains controversial (American Diabetes Ass. 1987, Canadian Diabetes Ass. 1981, Reaven 1980, Hollenbeck et al 1986). Interest in dietary management has intensified in recent years, associated with heightened efforts to normalize carbohydrate and lipoprotein metabolism in individuals with diabetes. However, an understanding of the impact that differences in dietary composition have on the metabolic abnormalities present within this syndrome is far from clear. The major emphasis in recent years has been on the reduction of total fat and saturated fat and replacement with complex carbohydrate. The rationale for this approach is based on the premise that such diets will reduce the risk of coronary artery disease by reducing total and LDL cholesterol concentrations. When the confounding factors are taken into consideration, there appears to be little evidence in support of view that substituting carbohydrate for fat in the diets of individuals with diabetes results in any measurable beneficial effect (Hollenbeck and Coulston 1991). It could be argued that the most characteristic defects in carbohydrate and lipoprotein metabolism are exacerbated in response to low-fat high-carbohydrate diets. Alternatively, the data suggest that diets containing conventional quantities of fat, in which saturated fat is replaced by unsaturated fat and dietary cholesterol reduced, would result in the desired reductions to total and LDL cholesterol concentrations without the adverse effects of increased postprandial glucose and insulin concentrations, increased fasting and postprandial total and VLDL triglyceride concentrations and decreased fasting HDL cholesterol concentrations (Colston et al 1989, Garg et al 1989).

Abbott and co-workers conducted two series of studies on individuals with obesity and/or non-insulin-dependent diabetes mellitus (NIDDM) to assess the effects of this dietary recommendation on both lipoproteins and their metabolism as well as on insulin secretion and action and energy expenditure (Abbott et al 1990a, 1990b). Both series compared a diet high in saturated fat with a diet high in complex carbohydrates and fiber. Calories and proportion of protein were constant. In the first set of studies, the aim was to examine the effect of replacement of saturated fat with complex carbohydrate in a regimen with conventional foods that would closely approximate foods expected to be used and recommended to diabetic patients. In the second regimen, more extreme difference between carbohydrate content and fat content was investigated using a dietary change that would approximate the contrasts between traditional diet of Native Americans or other cultures and a modern westernized diet.

The effects on lipoproteins included consistent decreases in total and LDL cholesterol (av 10%), minimum to no change in HDL cholesterol, and insignificant changes in total or VLDL triglycerides or 24-h triglyceride profiles. Changes in total and LDL cholesterol required 3-4 wk to reach equilibrium. Metabolic studies used to elucidate the reasons for the decrease in LDL cholesterol confirmed no stimulation of VLDL triglyceride or apolipoprotein B (apoB) production on the high-carbohydrate diet. The decrease in LDL appeared to be due to decreases in mechanisms that convert VLDL to LDL and increased activity of LDL apoB clearance. In the study with traditional foods, where dietary carbohydrate was 70% and fat only 15%, there was no improvement in glucose tolerance. It was accompanied by an improvement in glucose-mediated glucose disposal and insulin secretion. In individuals having a wide range of obesity and glucose tolerance, substitution of complex carbohydrates for saturated fat has beneficial effects of lowering LDL cholesterol and possibly improving glucose tolerance and insulin secretion but without having any adverse effects on lipoprotein metabolism or energy expenditure (Howard et al 1991).

In the earlier stages of progression of this disease, insulin resistance prevails, especially because most patients are overweight; β-cell function, while probably impaired, remains relatively responsive. However, because most of these patients are overweight, the primary aim of dietary therapy is to restrict total calories to achieve weight reduction. Thus, regardless of the composition of the diet, all nutrients (fat, carbohydrate and protein) are restricted. If we assume that the average obese patient with IGT is consuming 3000 kcal/day, for the purpose of weight reduction, and 1800-kcal diet can be recommended (Grundy 1991). Even if this diet has a high percentage of total fat (40%) and is relatively enriched in monounsaturated fatty acids (26%), the total fat will still have to be reduced from 133 to 80g/day. Therefore, on the weight-reduction diet, even monounsaturated fatty acids are curtailed. If a 25%-fat diet is used for weight reduction, total fat is cut further to 50g/day. The difference in fat intake between these two weight-reduction diets is equivalent to ~ 2 tbl an oleic-rich oil (e.g., olive oil or canola/rapeseed oil) or ~ 270 kcal/day from fat. There would appear to be little difference between these two 1800-kcal diets for obese patients with IGT, and personal preference may determine which diet to use.

A one-year randomized clinical trial of non-pharmacologic treatment of nondiabetic adults with hyperinsulinemia was recently carried out (Nilsson, et al, submitted). One group of subjects received intensive lifestyle intervention and the other was followed passively. Those in the active treatment were seen in groups of approximately 8 subjects. Each group met monthly at evening sessions, with the guidance of a group leader (nurse, dietician or physiotherapist). Each session lasted 1-2 hours, and participants were encouraged also to meet between sessions. On four occasions special physical activity (outdoor walking for two hours) was offered to all participants and their relatives. Special physical activity sessions were also offered biweekly to the most sedentary subjects. Participation in other activites of sport organizations were strongly recommended when appropriate. Special guided tours in local supermarkets, cooking demonstrations, and distribution of free olive oil were used to promote adherence to a healthier lifestyle. Group dynamic techniques were used to help participants join together and encourage each other.

A prudent low-fat, high-fiber diet was recommended, and dieticians performed four-day dietary recordings, at start and after one year, for evaluation of personal eating habits, as well as for individual counselling. A computer program for nutrients was used. Dietary recommendations included total fat <30% of daily energy intake, a high P/S ratio (0.8-1.0), and an increased consumption of mono- and polyunsaturated fatty acids. Intake of cholesterol was <200 mg/day. Dietary fiber was recommended at 30 g/day, mostly of the water-soluable type in oat and bean products, fruits, and vegetables. Fatty cold-water fish (salmon, mackerel) was recommended, and dietary salt reduced. Caloric intake was reduced in those with body mass index 27 kg/m^2^ and abdominal obesity (waist/hip 0.95). Physical activity was individually encouraged, based on improvements in ordinary life habits (walking, cycling, gardening). Aerobic activities of a dynamic, submaximal and long duration were preferred. Alcohol was accepted in reasonable amounts but overconsumption was discouraged.

In the active group, mean body weight decreased by 2.2 kg during the year, compared with a decrease of 0.1 kg in the passive group. Blood pressure, fasting plasma insulin and C-peptide concentrations also fell significantly in the intervention group only. In contrast, two-hour post-load blood glucose increased in the passive group but did not change significantly in the intervention group. Thus this study demonstrates the feasibility of the type of diet-exercise intervention proposed in this application, and its effect on body weight and glucose and insulin metabolism in nondiabetic Swedish subjects.

2.3.2 Justification for performance in Finland. The proposed study could potentially be conducted in many different locations, but there are several distinct advantages of performing the study in Finland by the applicant investigators:

1) The success of such therapy appears to be higher in Finland than elsewhere (e.g. England or North America), perhaps because of differences in social structure, with much more willingness to conform to prescribed behavior.

2) It is relatively easy in Finland to organize population screening programs and follow persons long term because of the universal use of personal identification numbers coupled with access to names, addresses, and vital status, and because of the social attributes referred to above.

3) Among the group of investigators, there is considerable expertise and experience not only in the conduct of pharmacologic and behavioral clinical trials, but also in diabetology, epidemiology, and biostatistics.

Table 2.1 STUDIES OF THE CUMULATIVE INCIDENCE (CI) OF DIABETES IN IGT

Study Definition Treat- N at Followup CI 5yr CI^#^

(Reference) of IGT ment risk (years) (%) (%)

Whitehall, UK 2hr BG Yes/No^*^ 204 5 13 13

(Jarrett, 1979) =6.7-11.0

Malmöhus, Sweden local No 59 10 29 15

(Sartor, 1980) criteria

" " " Yes 147 10 12 6

" WHO No 22 10 32 16

" WHO Yes 25 10 20 10

Bedford, UK 2hr cap BG Yes/No^*^ 241 10 15 8

(Keen, 1982) =6.7-11.1

Nauru WHO No 51 6 25 21

(King, 1984)

Tokyo, Japan 2hr BG No 288 9 17 9

(Kadowaki, 1984) =7.2-13.3

Israel NDDG criteria No 83 3 11 18

(Modan, 1986) for nondiag-

nostic or IGT

Pima Indians, USA WHO No 384╪ 5 25 25

" " " " " 10 61

(Saad, 1988a)

Malmö, Sweden WHO No 56 5 21 21

(Eriksson, in press) " Yes 161 5 11 11

Malta

(Schranz 1989) WHO No 75 6 31 26

San Antonio, USA WHO No 61 8 19 12

(Haffner, 1990)

Finland WHO No 234 5 12 12

(Stengård 1992)

^#^Estimated 5-year cumulative incidence by linear interpolation from actual follow-up period and cumulative incidence.

^*^In these clinical trials those subjects treated or not treated with hypoglycemic drugs were pooled as this treatment variable had no significant effect on the outcome.

╪Number at baseline, follow-up period varied between subjects.

CHAPTER 3. STUDY DESIGN

3.1 STUDY PURPOSE AND DESIGN SUMMARY

3.1.1 Purposes. The Diabetes Prevention Study (DPS) has three major purposes:

1) to assess the efficacy of a special diet-exercise program in preventing or delaying the onset of type 2 diabetes in persons with IGT, and

2) to study the risk factors for progression to diabetes in persons with IGT.

3) to assess the effects of the intervention on atherosclerotic vascular disease.

3.1.2 Primary Endpoint. The primary endpoint is diabetes, as defined in detail in Chapter 7.

Subjects will remain in the study and be followed until the end of the study or death, whichever occurs earlier. Study treatments will be continued until the end of the study, death, or the occurrence of diabetes, whichever occurs earlier.

3.1.3. Secondary endpoints. Subjects will also be monitored for the following secondary endpoints as defined in detail in Chapter 7:

1) Fatal or non-fatal myocardial infarction or sudden cardiac death.

2) Angina pectoris.

3) Intermittent claudication.

4) Stroke.

5) Death from any cause.

3.1.4 Subjects. Subjects with IGT will be found by population screening. A total of 560 subjects at several Clinical Centers will be treated and followed for at least five years. Each subject will be randomly allocated to a special or standard diet-exercise program. A Central Laboratory will make biochemical measurements, and a Coordinating Center will coordinate study activities and provide central data management and statistical support.

3.2 SUBJECT ELIGIBILITY AND EXCLUSION CRITERIA

3.2.1 Definitions of diabetes and IGT. The classification of and diagnostic criteria for diabetes and other categories of glucose tolerance will be based on criteria adopted by the World Health Organization (WHO), (World Health Organization, 1985). According to WHO criteria, the diagnosis of diabetes may be established on the basis of:

1) casual plasma glucose concentrations ≥ 11.1 mM (200 mg/dl) on one occasion with symptoms or on two without,

2) fasting venous plasma glucose concentrations ≥ 7.8 mM (140 mg/dl) on more than one occasion, or

3) oral glucose tolerance tests meeting the criteria shown in Table 3.1. IGT is diagnosed when the criteria for diabetes are not met, but the 2 hour post‑load plasma glucose is from 7.8 to 11.0 mM (140 to 199 mg/dl).

TABLE 3.1. DIAGNOSTIC VALUES FOR THE ORAL GLUCOSE TOLERANCE TEST

ACCORDING THE WORLD HEALTH ORGANIZATION (WHO)

Venous plasma glucose concentration

mM mg/dl

IGT Fasting < 7.8 <140

and 2 hour 7.8‑11.0 140‑199

Diabetes Fasting > 7.8 >140

or 2 hour >11.1 >200

3.2.2 Eligibility and exclusion criteria. To be eligible for

participation in the DPS, subjects must meet the following eligibility criteria. Subjects will have IGT, will be aged 40-64 years, and will not have a history of diabetes. The full eligibility and exclusion criteria are given in Section 5.1.

3.3 SCREENING FOR IGT

The screening procedure is described in Section 5.2.

3.4 ELIGIBILITY CRITERIA

There will be a comprehensive examination to determine eligibility.

To remain eligible, a subject must meet no further exclusion criteria upon review of results of the medical evaluations performed at the screening examinations (Section 5.3).

A subject not meeting the eligibility criteria will be discharged from the study, after being told that he/she is ineligible because of medical contraindications. All such exclusions and their reasons will be recorded as part of the study data.

3.5 REGISTRATION, RANDOMIZATION, AND STRATIFICATION

3.5.1 Treatment assignment and registration. Assignment will be issued by the Clinical Center which will register enrollment of subjects upon verification that all eligibility criteria have been met.

3.5.2 Randomization to treatment. Subjects will be randomly allocated to special or standard diet-exercise program in the following way:

Group 1: Special diet - exercise

Group 2: Standard diet - exercise

3.5.3 Stratification. Randomization will be stratified by 1) Clinical Center and 2) the 2-hour post-load glucose obtained during the OGTT:

o Stratum 1: venous plasma glucose = 7.8-9.4 mM (140-169 mg/dl)

o Stratum 2: venous plasma glucose = 9.5-11.0 mM (170-199 mg/dl)

3.5.4 Registration. Subjects are enrolled in the DPS as soon as they are registered by the Clinical Center. The Clinical Center will promptly register a subject upon verification that all eligibility criteria have been met. A registration number (and treatment assignment) is given to the subject according to the randomization list.

3.6 FOLLOW-UP

Subjects will be enrolled over a period of two years and followed for five or six years.

Follow-up visits will be scheduled with broad time windows (see Chapter 4) as follows: monthly intervals for the first 3 months and every 3 months for the remainder of the study.

Testing for diabetes and the secondary endpoints will be performed yearly during the treatment period with no interruption of treatment. The goal is to study the effects of the treatments as carried out continuously. It could be argued, however, that a treatment might be effective in controlling diabetes so that it cannot be diagnosed by OGTT, as opposed to preventing the disease. We do not believe this represents a major problem in interpretation because even very effective therapy of type 2 diabetes rarely results in normalization of the OGTT. Furthermore, we would consider suppression of diabetes to the point of undectability (and/or prevention of the secondary endpoints) to be successful effects of the treatments, even while the treatments were continued.

The question of control vs. prevention will be further considered in those who survive the full treatment period without developing diabetes. These subjects will be followed for the development or appearance of diabetes for an additional six months after termination of all active treatment.

The variables to be measured at follow-up are described in Chapter 5, and the endpoints to be monitored are described in Chapter 7.

3.7 MASKING

3.7.1 The diet-exercise intervention will be only partly masked. This assignment can obviously not be masked from the subjects or from those Clinical Center personnel who counsel the subjects on their diet or exercise programs. However, all laboratory personnel, including those performing the glucose tolerance test, and the Endpoints Committee (Chapter 8) will be masked.

3.8 SAMPLE SIZE AND ENROLLMENT GOAL

We begin sample size determination by estimating the 5-year incidence of diabetes in the untreated persons with IGT to be 28% (see Chapter 2). Because of the uncertainty in the data needed to make this estimate, this figure may be an underestimate of the incidence of diabetes. If so, the power of the study would be greater than the following computations indicate. We are designing the study to be large enough to be able to detect, with 80% power (beta = 20%) at the 2-tailed 5% significance level (alpha = 5%), a 30% reduction in diabetes incidence due to diet-exercise treatment, ie, a reduction from 28% to 19.6%.

The possibility of a reduction in diabetes incidence by at least 30% is suggested by the results of the Malmöhus study (Sartor, et al, 1980) analyzed in accordance with randomized treatment assignment and by the nonrandomized treatment study of Eriksson and Lindgärde (1991). We are assuming that noncompliance in the DPS will be no worse than in these previous studies; therefore we are making no further adjustment for noncompliance. We will, however incorporate an adjustment for losses to follow-up, including deaths.

Letting p_1_ and p_2_ denote, respectively, the proportion of events (incidence of diabetes) in the control and treated groups, respectively, then, according to Halperin, et al (1968), the sample size required in each of the two groups is

N = {Z_α_[2p(1 - p)]^1/2^ + Z_ß_[p_1_(1 - p_1_) + p_2_(1 - p_2_)]^1/2^}^2^ / [p_1_ -p_2_]^2^,

where p = (p_1_ + p_2_)/2 and Z_α_ and Z_ß_ are the normal deviates associated with α and ß. With p_1_ = .28, p_2_ = .175, α (2-tailed) = .05, and ß = .20, the total minimum required sample size becomes

2N = 500.

In Malmöhus County from 1962-87, the mortality rate among persons aged 45-64 with IGT by WHO criteria was 19/1000 persons per year (Knowler, et al, in preparation), yielding an estimated 5.5-year cumulatie mortality rate of 10% in study participants. Allowing for 10% losses to follow-up from deaths over a 5.5-year period increases the sample size to

2N = 500/(1 - .10) = 556, or approximately 560.

Because the prevalence of IGT in the study populations is not precisely known, the expected number of cases of IGT to be detected on screening can only be approximated and these estimates need to be confirmed in a pilot study. We are therefore specifying that the Clinical Centers should recruit a minimum of 560 subjects. In that the promise of an intervention program may be used in motivating recruitment, each center may recruit more than its minimum goal. Any total larger than 560 will serve to increase the study's power.

To study serial changes in carotic artery diameter and thickness of the intima and media by B-mode ultrasound much smaller sample size would be sufficient, because these ultrasound measurements are based on continuous variables and because the measurement variability is relatively low (Bond et al 1991). However, this proposed sample size is too small to be able to detect possible differences in the incidence of acute CHD and stroke events between the groups. On the other hand, these acute CVD events can be pooled with the findings from the ultrasound measurements of carotic arteries when assessing the rate of worsening of atherosclerosis.

3.9 PRETEST OF PROCEDURES AND DATA FORMS

During the 2-year screening period the DPS investigators will review the study design and detailed provisions of the Manual of Operations and agree on any revisions that seem appropriate. Data forms will be developed and the Policy and Safety Committee will meet for the purpose of reviewing and approving the Manual of Operations. The DPS investigators will then conduct a pretest of study procedures and data forms.

For the pretest, each Clinical Center will select 3 subjects who are found not to have IGT on screening. These subjects will undergo as many of the DPS study procedures as may be appropriate, eg, medical history, blood pressure, anthropometric, and laboratory measurements. Upon completion of the pretest, the procedures and data forms will be revised as appropriate.

CHAPTER 4. VISIT AND DATA SCHEDULE

4.1 OVERALL SCHEDULE

The schedule of examinations is summarized in Table 4.1. The starting date for enrolling subjects in the randomizized treatment period is designated as the beginning of year 1 of the study. Recruitment and enrollment should be completed by the end of year 2, i.e., two years later. All subjects will complete the study during the seventh year of the study. The closing date for each subject enrolled during the first year of the study will be the 6th anniversary of his/her randomization. The closing date for each subject enrolled in the second year will be the 5th anniversary of his/her randomization. Thus each subject will have either 5 or 6 years of follow-up, and follow-up of all subjects will be completed during one year.

4.2 SCHEDULED VISITS

Complete descriptions of the examinations at the visits are given in Chapter 5 and of the treatments in Chapter 6. Subjects will participate in the following visits (see Table 4.1):

4.2.1 Screening Visits. The screening OGTTs, eligibility measurements and medical history are performed at these 2 visits.

4.2.2 Enrollment Visit.

At this visit there is a physical examination by a physician for determination of eligibility. If the subject is eligible, clinical and biochemical measurements and randomization take place. The subject is counseled on the standard or special diet-exercise according to the random assignment.

4.2.3 Interim Visits. These visits occur at about 1-month intervals after enrollment for 3 months and at 3-month intervals for the remainder of the five or six years of follow-up. The visits occurring at annual intervals after enrollment are designated Annual Visits. Subjects receive diet-exercise counseling at interim visits according to the random assignment.

4.2.4 Annual Visits. At each of the five or six Annual Visits all biochemical tests, including the OGTT, are performed, the clinical measurements are made, and subjects receive diet-exercise counseling according to the random assignment.

4.3 TIME WINDOWS

For each visit, a time window is specified (Table 4.1). If the visit fails to occur within the time window, then it is a missed visit.

Table 4.1 FLOW SHEET FOR DATA COLLECTION

Clinical

Months & bio- Diet &

from History & chemical Diet & Exerc. Time window

enroll- Name Exam by measure- Exercise counsel- (y = years)

ment of visit OGTT Physician ments^1^ History ing^2^ and   (d = days)

monitoring

-2 1st Screening +

-1 2nd Screening + + +

0 Enrollment + + +

1 Interim + 16- 45 d

2 Interim + 46- 75 d

3 Interim + + 76-106 d

6 Interim + + 167-227 d

9 Interim + + 289-319 d

12 Annual + + + + + 320-410 d

15 Interim + + 1 y + 46-136 d

18 Interim + + 1 y + 137-227 d

21 Interim + + 1 y + 228-318 d

24 Annual + + + + + 1 y + 319-410 d

27 Interim + + 2 y + 46-136 d

30 Interim + + 2 y + 137-227 d

33 Interim + + 2 y + 228-318 d

36 Annual + + + + + 2 y + 319-410 d

39-60 or -72^3^

End of treatment+3^4^ + +

End of treatment+6 + +

^1^ See Chapter 5.

^2^ For subjects assigned to intensive diet-exercise, additional group sessions will occur, and for some subjects additional visits with dietary and exercise personnel will be required, especially during the first year. For subjects assigned to the standard diet-exercise program, counseling will be given only at the enrollment visit.

^3^ Months 39-60 (for those followed 5 years) or 36-72 (for those followed 6 years) will be the same as months 27 through 36 except for the yearly increments of the time windows.

^4^ See Section 5.5.

CHAPTER 5. ELIGIBILITY, SCREENING, AND EXAMINATION PROCEDURES

5.1 ELIGIBILITY AND EXCLUSION CRITERIA. To be eligible for

participation in the DPS, subjects must meet the following eligibility

criteria.

5.1.1 Eligibility criteria

o **Impaired glucose tolerance defined as follows**:

**a**) the mean of 2-hour plasma glucose of two OGTTs >7.8 and <11.0. If the mean is <7.8, and the 2-hour value of the first OGTT is >7.8 and the 2-hour value of the second OGTT is <7.8 and the fasting plasma glucose of the second OGTT is >5.5, then a third OGTT is performed and the mean 2-hour glucose is counted for the first and third OGTT

**OR**

**b**) one OGTT with fasting plasma glucose 6.4-7.7 and 2-hour value 7.8- 11.0

**OR**

**c**) random plasma glucose value after at least 4 hour fast 6.4-7.7 (or capillary blood -> 5.6-6.4) and one OGTT with 2-hour value 7.8-11.0.

o **Obesity, defined by body mass index >25 kg/m^2^**

o **Age 40-64 years**

o Willingness, after receiving a thorough explanation of

the study, to participate. Subject indicates willingness by signing the consent form.

5.1.2 Exclusion criteria

o Previous diagnosis of diabetes other than gestational diabetes (diabetes first diagnosed during a pregnancy) which has not persisted after pregnancy

o Previous treatment for IGT other than with routine dietary and health advice (persons previously given intensive diet or exercise treatment for IGT or other metabolic conditions such as hyperlipidemia will be excluded)

o Participation in a regular program of vigorous physical exercise

o A myocardial infarction or a by-pass operation less then 1 year ago o Hypersensitivity to diet or exercise program

o Chronic illness that makes 6-year survival improbable, or illness which requires or is likely to require treatment known to affect glucose tolerance (e.g. corticosteroid drugs)

o Mental or medical characteristics that are likely to interfere with informed consent, compliance with diet, exercise, or long-term follow-up

These eligibility and exclusion criteria are specified for the following reasons. The age range of 40-64 years is chosen to include a target population old enough for IGT to have a substantial prevalence to make screening feasible and young enough that the mortality rate will not cause many censored observations when development of diabetes is the primary endpoint. Pregnancy is to be avoided because of its effects on glucose tolerance and interference with treatment procedures. Subjects must be overweight and not participating in a vigorous exercise program, because weight loss and exercise will be components of the diet-exercise intervention. As first occurrence of diabetes will be the primary endpoint, it must not already have been diagnosed at entry. Previous treatment of IGT (such as by diet or exercise) may have established permanent changes in behavior which would make randomization to the standard diet-exercise group impractical. Entry of patients treated with drugs known to affect glucose tolerance would confuse interpretation of findings. The other medical contraindications are to avoid randomizing patients who would be unlikely to be able to maintain randomized treatment throughout the study.

Because diet is generally shared among members of a household, the enrollment of more than one person from the same household would compromise the independence of individual treatments. But since there are also benefits in enrolling both the husband and wife, they can be enrolled, but only the first one of them is randomized according to the randomization list and the spouse is assigned to the same treatment group.

The use of thiazide diuretic or beta blocker may affect glucose tolerance. Where appropriate, treatment may be changed to an ACE inhibitor or a calcium antagonist. If the subjects can not or does not want to change the medication, the subject can be eligible with the understanding that this drug treatment will not be altered without a major indication.

5.2 SCREENING FOR IGT

5.2.1 Population selected for screening. Cases of IGT to be studied will be found by population screening. The type of population screened will vary from one Clinical Center to another; some will select a sample representative of the general population while others will select subjects thought to be at high risk of IGT (eg, subjects who are obese, have hypertension, or have relatives with type 2 diabetes).

5.2.2 Conditions for the screening examination. Prior to the screening examination, any acute intercurrent illness or injury must be resolved and treatment of any chronic condition (such as hypertension, arthritis or hyperlipidemia) must be stabilized so that the test is performed under stable conditions.

5.2.3 The oral glucose tolerance test.

The OGTT should be administered in the morning after at least three days of unrestricted diet and usual physical activity. The test should be preceded by an overnight fast of 10-16 hours, during which water may be drunk. Smoking is not permitted during the test. The presence of factors that influence interpretation of the results of the test must be recorded (e.g., medications, inactivity, infection, etc.). In the first screening OGTT blood glucose may be analysed from capillary whole blood with a Hemocue-analyser, which is fast, easy, cheap, and yet reliable method in blood glucose screening.

After collection of the fasting venous blood sample (or capillary blood sample in the first OGTT), the subject should drink 75 g of anhydrous glucose (or partial hydrolysates of starch of the equivalent carbohydrate content or 82.5 g glucose monohydrate) in 250-300 ml of water over the course of 5 minutes. Venous (or capillary) blood samples will be collected at 1 and 2 hours after the test load (only at 2 hours in the first screening OGTT).

The blood sample should be collected in a tube containing NaF and centrifuged within 15 minutes to separate the plasma; the plasma should be kept at 4^o^C up to 12 hours or frozen until the glucose concentration is measured. In addition, at fasting a blood sample will be collected in a tube containing EDTA for measurement of HbA1c by high pressure liquid chromatography (HPLC).

Subjects with previously known IGT can be tested by this proce­dure to determine if they still have IGT, but must be excluded if they have been actively treated for IGT (see Section 5.1.2).

The test will be interpreted and acted on as follows:

1. Subjects with 2hr plasma glucose (or capillary whole blood glucose) <7.4 mM (<133 mg/dl) will be told that they have normal glucose tolerance, are ineligible for the study, and that no further action is indicated.

2. Subjects with 2hr plasma (or capillary whole blood) glucose 7.4‑11.3 mM (133-199 mg/dl) are asked to come to another OGTT after at least one week. Subjects with mean 2hr glucose 7.8-11.0 of two OGTTs are

diagnosed as having IGT and are further evaluated for eligibility to partic­ipate in the DPS.

3. Subjects with mean 2hr glucose >11.1 mM (>200 mg/dl) are diagnosed as having probable diabetes and are referred to the usual source of medical care for confirmation and treatment of diabetes.

5.3 SCREENING EXAMINATIONS

5.3.1 Conditions for examination are discribed in section 5.2.2.

5.3.2 Content of first screening examination.

This examination will include an OGTT and the following measurements

(1) Height (to the nearest cm without shoes).

(2) Weight (to nearest 0.1 kg in light indoor clothes, without shoes).

5.3.3 Content of second screening examination.

This examination will include an OGTT. Previous diagnoses or treatment for diabetes, IGT, or gestational diabetes will be sought. If present then the patient is ineligible for inclusion in the trial (see 5.1.2). Treatment for hypertension, hyperlipidemia, other serious illnesses and use of medicines, alcohol and tobacco will be recorded. The following measurements are made

(3) Measurement of waist and hip circumferences.

(4) Measurement of sagittal and transverse diameters.

(5) 12-lead supine resting ECG (to be forwarded to the ECG Reading Center).

5.3.3 Content of enrollment examination. This examination will include a medical history and physical examination by a physician.

The following core data will be obtained and recorded:

(6) Blood pressure measured with a mercury sphygmomanometer (1st and 5th phase).

(7) Fasting venous blood specimens for serum total and HDL cholesterol, serum triglycerides, serum creatinine, liver function tests (serum bilirubin, alkaline phosphatase, albumin, and ALT or AST), thyroid stimulating hormone, HbA1c by HPLC, and an extra 10 ml of serum to save frozen.

(8) HLA-typing and storage of DNA samples for genetic markers.

(9) WHO cardiovascular questionnaire will be completed.

(10) Subjects will be asked to complete a dietary diary.

(11) Physical activity (occupational and leisure) will be assessed by questionnaire.

(12) Assessment of physical fitness by walking test.

(13) B-mode ultrasound imaging of carotic arteries.

(15) Lean body mass using Futrex infrared equipment.

(16) Hemostatic factors: The activity of the hemostatic system, and its contribution to the progression and complications of atherosclerosis will be assessed by measuring fibrinogen, F VII:C, prothrombin fragment F_1+2_ and PAI-1 activity both **in the beginning and at the end of the study**.

5.4 ANNUAL EXAMINATIONS

5.4.1 Conditions for examination. Any acute intercurrent illness or injury should be resolved before the examination is carried out. The annual examination will be undertaken at each anniversary of randomization.

5.4.2 Content of annual examinations. The examination will include an OGTT and a medical history and physical examination by a physician. If the OGTT is diabetic, it is repeated after 1 week. If also the second OGTT is diabetic, the subject has reached the study endpoint.

(1) Any alteration in health status since the previous annual examination will be recorded. If a diagnosis of myocardial infarction, angina or diabetes has been made in the preceding year, then a copy of the clinical case notes must be forwarded to the Endpoints Committee.

(2) Treatment for hypertension, hyperlipidemia, other serious illnesses and use of medicines, alcohol and tobacco will be recorded.

(3) All of the core data described under the screening and enrollment examinations will be collected annually (excluding ultrasound measurements and genetic markers).

(4) B-mode ultrasound imaging of carotic arteries will be repeated at the last visit.

5.5 POST-STUDY FOLLOW-UP

All active treatments will cease at the end of 6 years (for those randomized in the first year of the study) or 5 years (for those randomized in the second year). The treatment will conclude with the usual annual examination (including an OGTT, Section 5.4). Thereafter, the reinforcement for the intensive diet-exercise program will be discontinued, although the patients assigned to this treatment will not be discouraged from continuing it on their own. All subjects who have not already reached the diabetes endpoint will be retested by OGTT three months and six months after termination of treatment. If one of these OGGTs indicates diabetes, the test will be repeated for confirmation of the diagnosis as described in Section 6.4.2. Following the six month OGTT, all procedures as specified by this protocol terminate, and further advice to subjects will depend on the outcome of the study.

CHAPTER 6. TREATMENT AND TESTING PROCEDURES

6.1 DIET-EXERCISE TREATMENT

Combined diet-exercise treatment will be compared to standard diet-exercise group. The groups will differ in several respects, including diet, physical activity, and weight loss.  Their comparison is intended to provide evidence as to which compo­nents affect the endpoint, but the main question is whether the combined treatment regimen is effective.

6.1.1 Standard diet-exercise treatment group. Subjects will be advised at entry to the trial by the dietitian to consume a diet with <30% of calories derived from fat, and to reduce alcohol and tobacco consumption to whatever extent possible.  Subjects should be advised to consume at least 15% of the daily calories at breakfast (defined as a meal taken within two hours of arising). Subjects will be advised to adjust total calories with the goal of weight loss to achieve body mass index <25 kg/m^2^.

These dietary goals will be implemented only by verbal or written instructions from a dietitian at the enrollment and annual visits.  None of the additional measures described below will be used.

6.1.2 Special diet-exercise treatment group.

(1) Subjects will be advised by the dietitian to consume a diet with ≥50% of calories derived from carbohydrate, 30-35% from fat (10 E% saturated fat, 20 E% unsaturated fat), cholesterol <300 mg/day, and 0.8 g protein/kg body weight/day. Dietary fiber will be increased to 30 g/day, or more if tolerated. This goal may be achieved through use of natural high fiber foods and by supplementation by soluble fibers such as beet fiber (Hagander, et al, 1988b). The caloric content of any supplemental fibers will be taken into account in the total caloric content of the diet. If subjects do not tolerate this much dietary fiber, the amount should be reduced to a tolerable level rather than withdrawing the subject from the diet treatment. Subjects will be advised to consume at least 10% of the daily calories at breakfast. Subjects will be encouraged to reduce alcohol and tobacco consumption.

(2) Intensive weight loss advice should be provided aiming to reduce body mass index to <25 kg/m^2^. If the subject does not succeed to lose weight during the first 6 months of the study and if his body mass index is >30, then VLCD (very low calorie diet) treatment may be used.

(3) Subjects will receive frequent regular dietary advice (see Table 4.1 for scheduled visits to the dietitian).

(4) Subjects will be advised to increase their level of physical activity by engaging in a walking program and will be given other advice on increasing leisure activities aiming at increasing the lean body mass and aerobic capasity.

(5) Frequent group meetings including diet and exercise activity and also aiming at improving the compliance will be held regularly throughout the study period.

6.2 MONITORING FOR COMPLIANCE AND ENDPOINTS

6.2.1 Interim visits. Interim monitoring will be for the primary purposes of improving compliance with the diet-exercise therapy. Interim visits will take place at 1, 2, and 3 months after randomization and thereafter every three months for the duration of the study. When this schedule coin­cides with the annual examinations (i.e. at months 12, 24, 36, 48, 60, and 72), the interim and annual examinations will be combined into a single visit.

At the interim visits, subjects will be seen by a dietitian or nurse for assessment of general health and measurement of body weight. No physical examination or laboratory tests will be performed unless a physician considers this to be indicated. Dietary, exercise, and weight loss counseling will be provided according to the assigned diet-exercise treatment.

The interim visits can be conducted in usual medical clinics, community centers, or patients' homes, as needed to ensure compliance.

6.2.2 Annual examination and OGTT.  The primary end point (diabetes) will be assessed annually as described under the Annual Examination (Section 5.4). The results of the OGTT at the annual exam should be interpreted as follows:

1. If the results are normal or indicate IGT (i.e. fasting plasma glucose <7.8 mM (<140 mg/dl) and 2hr plasma glucose <11.1 mM (<200 mg/dl), then continue the assigned treatments.

2. If the results are diagnostic of diabetes (i.e. fasting plasma glucose >7.8 mM (>140 mg/dl) or 2hr plasma glucose >11.1 mM (>200 mg/dl), then repeat the OGTT after one week. If the repeat test results are normal or indicate IGT, then resume the assigned treatments.  If they are diagnostic of diabetes, the subject has reached the study endpoint.

If at any time other than an annual evaluation the physician suspects, on the basis of signs or symptoms, that diabetes has developed in a patient, the patient should be evaluated as follows. If his/her condition permits, a fasting plasma glucose should be measured. If it meets the diagnostic criterion for diabetes (plasma glucose >7.8 mM (>140 mg/dl) and the patient's condition allows further testing, he/she should be evaluated as described for annual evaluations (i.e., an OGTT while continuing treatment, and if it is diagnostic of diabetes, a repeat test after seven days). If the patient's condition is urgent, a diagnosis of diabetes can be made by symptoms of diabetes and repeated fasting or casual plasma glucose concentrations >11.1 mM (>200 mg/dl). In this case, a standard OGTT as described under annual evaluations should be performed when and if the patient's condition improves sufficiently after standard treatment for diabetes and the fasting plasma glucose is <11.1 mM. If the fasting plasma glucose remains >11.1 mM after stabilization, no further OGTT is required. When the diagnosis of diabetes is confirmed, the subject has reached the study endpoint.

6.2.3 Diet-exercise treatment. Compliance with the diet-exercise program will be monitored by interviews with subjects by the dietitian at each clinic visit.

6.2.4. Physical activity assessment. The assessment of physical activity and cardiorespiratory fitness will be carried out using four different methods: (a) a questionnaire on 12-month leisure time physical activity history (Salonen and Lakka 1987, Lakka and Salonen 1992) (b) 24-hour total physical activity recording (Salonen and Lakka 1987, Lakka and Salonen 1992) (c) a 2-km walking test for assessing cardiorespiratory fitness as proposed recently (Kline et al 1987, Oja et al 1991). (d) assessment of maximal oxygen uptake form heart response to a given submaximal workload using a bicycle ergometer recorded at steady-state with a heart rate > 120 beats/min.

The measurements will be done before the randomization and on every annual visit. The walking test will also serve as an assessment of qualification of study subjects to the intervention program. If a person fails to complete the walking test he/she will not be recruited to the study.

Leisure time physical activity (LTPA) will be assessed by a 12-month leisure time physical activity history (Salonen and Lakka 1987, Lakka and Salonen 1992), modified from the Minnesota leisure time physical activity questionnaire. (Taylor et al 1978) The checklist in the 12-month history include the most common leisure time physical activities of middle-aged Finnish men, selected on the basis of a previous population study in Finland. (Mälkiä et al 1988) Leisure time physical activities are classified as conditioning and nonconditioning physical activity and walking and bicycling to and from work. Main categories of conditioning physical activity are 1. walking, 2. jogging, 3. skiing, 4. bicycling, 5. swimming, 6. rowing, 7. ball games, and 8. gymnastics, dancing, or weight lifting. Main categories of nonconditioning physical activity are 1. crafts, repairs, or building, 2. yard works, gardening, farming, or snow showeling, 3. hunting, picking-up berries, or cathering mushrooms, 4. fishing, and 5. forest work or forestry. Walking and bicycling to and from work will be asked separately because these physical activities are common in Finland and they are generally less intensive than conditioning walking and bicycling.

In the 12-month history the subjects are asked to fill in the frequency (sessions/each month), the average duration (hours and minutes/session), and the intensity (scored 0=recreational, 1=conditioning, 2=brisk conditioning, and 3=competitive, strenuous exercise) of each physical activity performed during the previous 12 months. Missing data are completed by a trained interviewer. Each intensity class of each physical activity has its own metabolic unit (MET) value, which represents the intensity of the physical activity. The MET is the ratio of metabolic rate during physical activity to the metabolic rate at rest. One MET is approximately 1 kcal/kg/hour and corresponds an oxygen uptake of 3.5 ml/kg/minute. The MET-values for specific physical activities were revised on the basis of a synthesis of available empirical data. (Durnin and Passmore 1967, Fox et al 1972, Mälkiä et al 1988) The total duration of each physical activity is computed by multiplying the frequency and the average duration and the energy expenditure by multipying the total duration and the intensity by body weight in kilograms.

Total physical activity will be assessed by a 24-hour total physical activity recording. (Salonen and Lakka 1987, Lakka and Salonen 1992) Physical activities are classified according to their intensity, as expressed in METs, into eight groups. The subjects record the physical activities in periods of 30 minutes and total time spent in each physical activity during 24 hours is summed. Energy expenditure in each physical activity during 24 hours is computed by multiplying the duration and intensity of the physical activity by body weight in kilograms. Total energy expenditure in physical activity is computed by summing energy expenditure values of individual physical activities.

6.2.5 Assessment of diet. The assessment of diet will be carried out using a 3-day food diary. The members of the intervention group will keep food diary every three months; the members of control group annually.

CHAPTER 7. END-POINTS CLASSIFICATION

The primary end point for the trial is worsening from IGT to diabetes mellitus.

The secondary end points for the trial are:

(1) Definite myocardial infarction (fatal and non-fatal) and sudden cardiac death

(2) Angina

(3) Possible myocardial infarction

(4) Intermittent claudication

(5) Stroke.

(6) Atherosclerosis

7.1 WORSENING FROM IGT TO DIABETES

This will be defined by the results of an oral glucose tolerance test which is diagnostic of diabetes (i.e. fasting plasma glucose > 7.8 mM or 2 hr plasma glucose > 11.1 mM). Confirmation by a repeat OGTT after seven more days is required, as described in Section 6.4.2.

7.2 CARDIOVASCULAR END POINTS

7.2.1 Definite myocardial infarction:

(1) a definite ECG abnormality, or

(2) typical or atypical symptoms and cardiac enzymes that are elevated to more than twice the normal, or

(3) typical symptoms together with abnormal enzymes with an absent or non-codable ECG.

(4) fatal cases, whether sudden or not, with naked eye appearance of fresh myocardial infarction and/or recent coronary occlusion found at necropsy (ante-mortem thrombus, hemorrhage into an atheromatous plaque or embolism).

7.2.2 Possible myocardial infarction: Typical symptoms without ECG changes or enzymes results that place them in the category definite myocardial infarction.

7.2.3 Ischemic cardiac arrest: Patients who were successfully resuscitated from a cardiac arrest but did not meet criteria for myocardial infarction.

7.2.4 Sudden cardiac death: Coronary heart disease stated on the death certificate and death known to have occurred within one hour after the onset of symptoms.

7.2.5 Unwitnessed cardiac death: Coronary heart disease stated on the death certificate with no evidence to justify a classification of sudden cardiac death.

7.2.6 Angina: Defined by a positive response to the WHO Questionnaire.

7.2.7 Intermittent claudication: Defined by a positive response to the WHO Questionnaire.

7.2.8 Stroke: Fatal stroke is defined by the End Points Committee after a review of all the available clinical documentation for subjects with a death certificate ICD code - (9th Revision). Documentation will be reviewed without knowledge of the subject's treatment. Non-fatal stroke is defined by a clinical diagnoses confirmed by the End Points Committee after review of all available clinical documentation.

7.2.9 Atherosclerosis: In addition to the "core" data mentioned above, measurements of the wall thickness (intima+media) of the carotid arteries will be performed at the first run-in examination using a non-invasive B-mode ultrasound technique. The measurements will be performed under rigorous standardization and quality control. The measurements in different centers will be recorded on video cassettes, which will be read centrally. The reading center will be blinded as to the treatment group of the participant. Ultrasound measurements of the distensibility of carotid arteries are also considered depending on the avaialability of suitable equipment and necesaary expertice. These measurements will be repeated at the end of study period (6th year) in order to get a quantitative assessment of the progression of atherosclerosis in different treatment groups.

7.3 END-POINT CLASSIFICATION

Minnesota coding will be performed by the ECG Reading Center. The End Points Committee will evaluate the classification of all end points with the exception of angina and intermittent claudication which will be determined by the response to the WHO Questionnaire. The committee will also review the documentation of diabetes diagnoses to confirm that this endpoint has been defined according to protocol. All end point classifications will be made without knowledge of the subject's treatment. Classification will not be altered once the treatment codes are broken.

CHAPTER 8. DATA ANALYSIS

8.1 INTRODUCTION

Data analysis in the DPS will be designed to meet the study's scientific objectives, recapitulated here:

1. To assess the efficacy of a diet-exercise program

in preventing or delaying the development of type 2 diabetes in persons with IGT.

2. To study the clinical course and prognosis of IGT.

3. To assess efficacy of the program in preventing atherosclerosis

The data developed in the DPS will provide a foundation for information not only on the treatment, but also on the natural history and prognosis of IGT with particular respect to the development of overt diabetes and macrovascular disease.

Presentations of DPS results will include the numbers of subjects on which the results are based as well as measures of sampling variability, such as standard errors or confidence intervals.

In addition to analyses directed at meeting the study's scientific objectives, there will also be analyses directed at monitoring data quality and the smooth and efficient operation of the study itself, such as analyses, by Clinical Center, to monitor progress in screening and subject enrollment, the completeness and internal consistency of data submitted to the Coordinating Center, delays in the submission of the data, and missed visits and study dropouts.

8.2 PRETREATMENT CHARACTERISTICS

An analysis of pretreatment characteristics of enrolled subjects, by Clinical Center and by assigned treatment, will be undertaken in order to provide descriptions of the population studied and to determine whether stratified randomization succeeded in balancing the various treatment groups with respect to potential risk factors for diabetes. In the unlikely event that such balance is not achieved, methods of covariate adjustment, such as standardization of rates or the Cox proportional hazards model will be used.

The pretreatment characteristics to be analyzed include:

o age, sex, ethnicity

o physical fitness

o physical activity

o waist and hip circumference, waist-hip ratio

o sagittal and transverse diameters

o height, weight, body mass index

o lean body mass

o systolic and diastolic blood pressure, hypertension

o cigarette smoking

o dietary intake

o laboratory measurements (plasma glucose and lipids, serum creatinine, c-peptide, and insulin)

8.3 OUTCOME VARIABLES

8.3.1 Events. The primary outcome variable is the incidence of diabetes. Other events of interest are defined in Chapter 7. The cumulative incidence of events, by treatment group and, to evaluate prognostic factors, by subgroups determined by pre-randomization characteristics such as those listed in Section 5.3.2, will be analyzed in 1-year follow-up intervals by the lifetable method.

8.3.2 Continuous variables. Mean changes in the following variables, by treatment group and subgroups defined by pre-randomization characteristics, will be analyzed in 1-year follow-up intervals:

o systolic and diastolic blood pressure

o waist-hip ratio; waist and hip circumference

o sagittal and transverse diameters

o body mass index

o lean body mass

o GTT

o serum cholesterol

o serum triglycerides

o serum creatinine

o plasma glucose

o plasma insulin

o serum c-peptide

o glycosylated hemoglobin

8.4 CLINICAL COURSE AND PROGNOSIS

In addition to the comparisons of outcomes between groups according to treatment assignment, clinical course and prognosis within treatment groups will be assessed; in this regard, the group assigned to the standard diet-exercise program will be of primary interest in that this group will provide information on the natural history of IGT.

Both univariate and multivariate (eg, logistic regression and proportional hazards) analyses, with the risk factors of Section 11.4 as independent variables and the events of Section 11.3 as dependent variables, will be attempted, as will lifetable analyses (eg, Kaplan-Meier) of events.

APPENDIX A

GLOSSARY

Diabetes. Defined in accordance with WHO criteria in Table 3.1.

Impaired glucose tolerance. Defined in accordance with WHO criteria in Table 3.1.

Type 1 diabetes. Insulin-dependent diabetes.

Type 2 diabetes. Non-insulin-dependent diabetes.

Missed Visit. Failure of a patient to appear for a Study Visit within the defined Time Window (Chapter 4).

Run-in period. Eight-week period preceding randomization during which subjects are assigned to 4 weeks of metformin and 4 weeks of placebo treatment (Section 3.4).

Study Visit. A regularly scheduled visit to an DPS Clinical Center by a DPS subject required for the completion of DPS procedures and DPS data forms (Chapter 4).

Time Window. Time interval within which a Study Visit must be completed (Table 4.1).

APPENDIX B

CENTERS AND INVESTIGATORS

Clinical Centers

Helsinki

Jaakko Tuomilehto, MD, PhD, Professor (Director)

Johan Eriksson, MD, PhD

Timo Valle, MD

Jaana Lindström, MSc.

Kuopio

Matti Uusitupa, MD, PhD, Professor

Anne Louheranta, MSc.

Turku

Helena Hämäläinen, MD, PhD

Aapo Lehtonen, MD, PhD, Professor

Tero Viljanen, MSc.

Tampere

Pirjo Ilanne-Parikka, MD

Oulu

Sirkka Keinänen-Kiukaanniemi, MD, PhD, Professor

Biochemistry Laboratory

National Public Health Institute Biochemistry Laboratory, Helsinki

Christian Ehnholm, Professor (director)

APPENDIX C

BIBLIOGRAPHIC REFERENCES

Abbott WGH, Howard BV, Rutolo G, Ravussin E: Energy expenditure in humans: effects of dietary fat and carbohydrate. Am J Physiol 1990;258:E347-351.

Abbot WGH, Swinburn B, Ruotolo G, Hara H, Patti L, Harper I, Grundy SM, Howard BV: Effect of a high carbohydrate, low saturated fat diet on apolipoprotein B and triglyceride metabolism in Pima Indians. J Clin Invest 1990;86:642-650.

American Diabetes Association: Nutritional recommendations and principles for individuals with diabetes mellitus: 1986. Diabetes Care 1987;10:126-32.

The ARIC Investigators. The atherosclerosis risk in communities (ARIC) study: design and objectives. Am J Epidemiol 1989;129:687-702.

Balkau B, King H, Zimmet P, Raper LR: Factors associated with the development of diabetes in the Micronesian population of Nauru. Am J Epidmemiol 1985;122:594-605.

Bitzén PO, Melander A, Scherstén B, Wåhlin-Boll E (1988): Influence of glipizide on early insulin release and glucose disposal before and after dietary regulation in diabetic patients with different degrees of hyperglycaemia. Eur J Clin Pharmacol 35:31-37.

Björntorp P: Abdominal obesity and the development of non-insulin-dependent diabetes mellitus. Diabetes Metab Rev 1988;4:615-622.

Björntorp P, Fahlen m, Grimby G, et al: Carbohydrate and lipid metabolism in middle-aged, physically well-trained men. Metabolism 1972;21:1037-1044.

Björntorp P, de Jounge K, Sjöström L, Sullivan L: Physical training in human obesity. II. Effects of plasma insulin in glucose-intolerant subjects without marked hyperinsulinemia. Scand J Clin Lab Invest 1973;32:41-45.

Black HR. The coronary artery disease paradox: The role of hyperinsulinemia and insulin resistance and implications for therapy. J Cardiovas Pharmacol 1990;15(Suppl. 5):S26-S38.

Bond MG, Barnes RW, Riley WA, Wilmoth SK, Chambless LE, Howard G, Owens B and the ARIC Study Group. High-resolution B-mode ultrasound scanning methods in the atherosclerosis risk in communities study (ARIC). J Neuroimag 1991;1:68-73.

Burstein R, Polychronakos C, Toews CJ, MacDougall JD, Guyda JH, Posner BI: Acute reversal of the enhanced insulin action in trained athletes: association with insulin receptor changes. Diabetes 1985;34:756-760.

Butler WJ, Ostrander LD, Carman WJ, Lamphiear DE: Diabetes mellitus in Tecumseh, Michigan: prevalence, incidence and associated conditions. Am J Epidmeiol 1982;116:971-980.

Canadian Diabetes Association: Special Report Committee guidelines for the nutritional management of diabetes mellitus: a special rport form the Canadian Diabetes Association. J Can Diet Assoc 1981;42:110-18.

Cassell GH, Ferris FL (1984): Site visits in a multicenter ophthalmic trial. Controlled Clin Trials 5:251-62.

Coulston AM, Hollenbeck CB, Swislocki AML, Reaven GM: Persistance of hypertriglyceridemic effect of low-fat high-carbohydrate diets in NIDDM patients. Diabetes Care 1989;12:94-101.

DeFronzo RA. The triumvirate: β-cell, muscle, liver: a collusion responsible for NIDDM. Diabetes 1988;37:667-687.

Durnin JVGA, Passmore P. Energy, work and leisure. London, Heinemann Educational Books LTD, 1967.

Eriksson K-F, Lindgärde F (1990): Impaired glucose tolerance in a middle-aged male urban population: a new approach for identifying high-risk cases Diabetologia 33:526-531.

Eriksson K-F, Lindgärde F: Prevention of type 2 (non-insulin-dependent) diabetes mellitus by diet and physical exercise: the six-year Malmö feasibility study. Diabetologia 1991;34:891-98.

Evans DJ, Hoffman RG, Kalkhoff RK, Kissebah AH: Relationship of body fat topography to insulin sensitivity and metabolic profiles in premenopausal women. Metabolism 1984;33:68-75.

Ferris FL, Ederer F (1979): External monitoring in multiclinic trials: applications from ophthalmologic studies. Clin Pharmacol Therapeutics 25 (No 5, Part 2):720-3.

Fox SM, Naughton JP, Gorman PA. Physical activity and cardiovascular health. III. the exercise prescription: frequency and type of activity. Mod Conc Cardiovasc Dis 1972;41:25-30.

Friedman LM, Furberg CD, DeMets DL (1985): Fundamentals of Clinical Trials. Second Edition. PSG Publishing Co, Littleton, Mass.

Fuller JH, Shipley MJ, Rose G, Jarrett RJ, Keen H (1980): Coronary-heart-disease risk and impaired glucose tolerance. Lancet 1:1373-6.

Garg G, Bonanome A, Grundly SM, Zhang Z-J, Unger RH: Comparison of a high-carbohydrate diet with a high-monounsaturated fat diet in patients with non-insulin-dependent diabetes mellitus. N Engl J Med 1988;319:829-34.

Goodyear LJ, Hirshman MF, King PA, Horton ED, Thompson CM, Horton ES: Skeletal muscle plasma membrane glucose transport and glucose transporters after exercise. J Appl Physiol 1990;68:193-198.

Haffner SM, Stern MP, Hazuda HP, Rosenthal M, Knapp JA, Malina RM: Role of obesity and fat distribution in non-insulin-dependent diabetes mellitus in Mexican Americans and Non-Hispanic whites. Diabetes Care 1986;9:153-161.

Haffner SM, Stern MP, Mitchell BD, Hazuda HP, Patterson JK (1990): Incidence of type II diabees in Mexican Americans predicted by fasting insulin and glucose levels, obesity, and body-fat distribution. Diabetes 39:283-288.

Hagander B, Asp N-G, Efendic S, Nilsson-Ehle P, Scherstén B (1988a): Dietary fiber decreases fasting blood glucose levels and plasma LDL concentration in noninsulin-dependent diabets mellitus patients. Am J Clin Nutrition 47:852-858.

Hagander B, Holm J, Asp N-G, Efendic S, Lundquist I, Nilsson-Ehle P, Scherstén B (1988b): Metabolic response to beet fibre test meals. J Human Nutrition and Dietetics 1:239-246.

Halperin M, Rogot E. Gurian J, Ederer F (1968): Sample sizes for medical trials with special reference to long-term therapy. J Chron Dis 21:13‑24.

Harris MI, Hadden WC, Knowler WC, Bennett PH (1987): Prevalence of diabetes and impaired glucose tolerance and plasma glucose levels in U.S. popula­tion aged 20-74 yr. Diabetes 36:523-534.

Helmrich SP, Ragland DR, Leung RW, Paffenbarger RS Jr (1991): Physical activity and reduced occurrence of non-insulin-dependent diabetes mellitus. New England Journal of Medicine 325:147-152.

Hirshman MF, Wardzala LJ, Goodyear LJ, Fuller SP, Horton ED, Horton ES: Exercise training increases the number of glucose transporters in rat adipose cells. Am J Physiol 1989;257:E520-E530.

Hollenbeck CB, Coulston AM, Reaven GM: Dietary carbohydrate: a different perspective. Diabetes Care 1986;9:641-47.

Hollenbeck CB and Coulston AM. Effects of dietary carbohydrate and fat intake on glucose and lipoprotein metabolism in individuals with diabetes mellitus. Diabetes Care 1991;14:774-785.

Horton ES: Role and management of exercise in diabetes mellitus. Diabetes Care 1988;11:202-11.

Horton E: Exercise and decreased risk of NIDDM. New Engl J Med 1991;325:147-52.

Horton ES: Exercise and physical training: effects on insulin sensitivity and glucose metabolism. Diabetes Metab REv 1986;2:1-17.

Jarrett RJ, Keen H, Fuller JH, McCartney M (1979): Worsening to diabetes in men with impaired glucose tolerance ("borderline diabetes"). Diabetologia 1979;16:25-30.

Joslin EP: The prevention of diabetes mellitus. JAMA 1921;76:79-84.

Kadowaki T, Miyake Y, Hagura R, Akanuma Y, Kajinuma H, Kuzuya N, Takaku F, Kosaka K (1984): Risk factors for worsening to diabetes in subjects with impaired glucose tolerance. Diabetologia 1984;26:44-49.

Keen H, Jarrett RJ, Fuller JH (1973): Tolbutamide and arterial disease in borderline diabetics. Proc VIIIth Congress IDF 1973;588-602.

Keen H, Jarrett RJ, McCartney P (1982): The ten-year follow-up of the Bedford Survey (1962-1972): Glucose tolerance and diabetes. Diabetologia 1982;22: 73-78.

King H, Kriska AM: Prevention of Type II diabetes by physical training; epidemiological considerations and study methods. Diabetes Care 1991 (in press).

King H, Taylor R, Koteka G, Nemaia H, Zimmet PZ, Bennett PH, Raper LR: Glucose tolerance in Polynesia: population-based surveys in Rarotonga and Niue. Med J Aust 1986;145:505-510.

King H, Zimmet P, Raper LR, Balkau B: The natural history of impaired glucose tolerance in the Micronesian population of Nauru: A six year follow-up study. Diabetologia 1984;26:39-43.

King H, Zimmet P, Raper LR, Balkau B: Risk factors for diabetes in three Pacific populations. Am J Epidemiol 1984;119:396-409.

Kline GM, Porcari JP, Hintermaister M, Freedson PS, Ward A, McCarron RF, Ross J, Rippe JM: Estimation of VO_2_ from a one-mile track walk, gender, age and body weight. Med Sci Ports Exerc 1987;3:253-259.

Knowler WC, Pettitt DJ, Bennett PH, Savage PJ: Diabetes incidence in Pima Indians: contributions of obesity and parental diabetes. Am J Epiemiol 1981;113:144-156.

Knowler WC, Sartor G, Scherstén B: Impaired glucose tolerance and mortality: a 22-year follow-up of tolbutamide treatment (in preparataion)

Krotkiewski M, Björntorp P: Muscle tissue in obesity with different distribution of adipose tissue: effects of physical training. Int J Obes 1986;10:331-341.

Lakka TA, Salonen JT. Physical activity and serum lipids: a cross-sectional population study in Eastern Finnish men. Am J Epidemiol 1992;136:806-18.

Little RR, England JD, Wiedmeyer H-M, Madsen RW, Pettitt DJ, Knowler WC, Goldstein DE: Glycosylated hemoglobin predicts progression to diabees mellitus in Pima Indians with impaired glucose tolerance (submitted)

Little RR, England JD, Wiedmeyer H-M, McKenzie EM, Pettitt DJ, Knowler WC, Goldstein DE (1988): Relationship of glycosylated hemoglobin to oral glucose tolernce: inplications for diabetes screening. Diabetes 37:60-64.

Manson JE, Nathan DM, Krolewski AS, Stampfer MJ, Willett WC, Hennekens CH. A prospective study of exercise and incidence of diabetes amond US male physicians. JAMA 1992;268:63-67.

Manson JE, Rimm EB, Stampfer MJ, Colditz GA, Willett WC, Krolewski AS, Rosner B, Hennekens CH, Speizer FE. Physical activity and incidence of non-insulin-dependent diabetes mellitus in women. Lancet 1991;338:774-778.

Medalie JH, Papier CM, Goldbourt U, Herman JB: Major factors in the development of diabetes mellitus in 10,000 men. Arch Intern Med 1975;135:811-817.

Meinert CL: Clinical Trials: Design, Conduct, and Analysis. New York, Oxford, 1985.

Modan M, Halkin H, Almog S, et al: Hyperinsulinemia: a link between hypertension obesity and glucose intolerance. J Clin Invest 1985;75:809-817.

Modan M, Karasik A, Halkin H, Fuchs Z, Lusky A, Shitrit A, Modan B (1986): Effect of past and concurrent body mass index on prevalence of glucose intolerance and type 2 (non-insulin-dependent) diabetes and on insulin response: The Israel study of glucose intolerance, obesity and hyper­tension. Diabetologia 1986;29:82-9.

Mowery RL, Williams OD (1979): Aspects of clinic monitoring in large-scale multiclinic trials. Clin Pharmacol Therapeutics 1979;25 (No 5, Part 2):717-9.

Mälkiä E, Impivaara O, Maatela J, Aromaa A, Heliövaara M, Knekt P. Physical activity of Finnish adults (In Finnish with English summary). Publications of the social Insurance Institute, Finland, ML: 80, Turku, 1988.

National Institutes of Health: Consensus development conference on diet and exercise in non-insulin-dependent diabetes mellitus. Diabetes Care 1987;10:639-644.

Nilsson P, Lindholm L, Scherstén B: Non-pharmacological treatment of hyperinsulinemia improves total cardiovascular risk profile: results from a one-year controlled trial (submitted)

O'Dea K: Marked improvement in carbohydrate and lipid metabolism in diabetic Australian aborigines after temporary reversion to traditional lifestyle. Diabetes 1984;33:596-603.

Ohlson LO, Larsson B, Björntorp P, Eriksson H, Svardsudd K, Welin L, Tibblin G, Wilhelmsen L: Risk factors for type 2 (non-insulin-dependent) diabetes mellitus: thirteen and one-half years of follow-up of the participants in a study of Swedish men born in 1913. Diabetologia 1988;31:798-805.

Oja P, Laukkanen R, Pasanen M, Tyry T, Vuori I: A 2-km walking test for assessing the cardiorspiratory fitness of healthy adults. Int J Sports Med 1991;12:356-362.

Peiris AN, Mueller RA, Struve MF, Smith GA, Kissebah AH: Relationship of androgenic activity to splanchnic insulin metabolism and peripheral glucose utilization in premenopausal women. J Clin Endocrinol Metab 1987;64:162-169.

Pignoli P, Temoli E, Poli A, Oreste P, Paoletti R. Intimal plus medial thickness of the arterial wall: a direct measurement with ultrasound imaging. Circulation 1986;74:1339-1406.

Pyörälä K, Laakso M, Uusitupa M. Diabetes and atherosclerosis: An epidemiologic view. Diabetes/Metab Rev 1987;3:463-524.

Reaven GM: How high the carbohydrates? Diabetologia 1980;19:409-13.

Riley WA, Barnes RW, Bond GM, Evans G, Chambless LE, Heiss G and the ARIC Study Group. High-resolution B-mode ultrasound reading methods in the atherosclerosis risk in communities (ARIC) cohort. J Neuroimag 1991;1:168-171.

Saad MF, Knowler WC, Pettitt DJ, Nelson RG, Bennett PH (1988b): Transient impaired glucose tolerance in Pima Indians: is it important? Br Med J 1988;297:1438-1441.

Saad MF, Knowler WC, Pettitt DJ, Nelson RG, Mott DM, Bennett PH (1988a): The natural history of impaired glucose tolerance in the Pima Indians. N Engl J Med 1988;319:1500-1506.

Salonen JT, Lakka TA. Assess­ment of physical activity in popula­tion studies - va­lidity and consis­tency of the methods in the Kuopio Ischaemic Heart Disease Risk Factor Study. Scand J Spo­rts Sci 1987;9:89-95.

Sartor G, Scherstén B, Carlström S, Melander A, Nordén Å, Persson G (1980): Ten-year follow-up of subjects with impaired glucose tolerance: preven­tion of diabetes by tolbutamide and diet regulation. Diabetes 1980; 29:41-4.

Schranz A, Tuomilehto J, Marti B, John Jarrett R, Grabauskas V, Vassallo A: Low physical activity and worsening of glucose tolerance: results from a 2-year follow-up of a population sample in Malta. Diabetes Res Clin Pract 1991;11:127-136.

Shimokata H, Anders R, Coon PJ, Elahi D, Muller DC, Tobin JD: Studies in the distribution of body fat. II. Longitudinal effects of change in weight. Int J Obes 1989;13:455-464.

Simpson HCR, Simpson RW, Lousley S, Carter RD, Geekie M, Hockaday TDR, Mann JI (1981): A high carbohydrate leguminous fibre diet imporves all aspects of diabetic control. Lancet 1981;I:1-5.

Stanhope JM, Prior IAM: The Tokelau island migrant study: prevalence and incidence of diabetes mellitus. NZ Med J 1980;92:417-421.

Stengård J, Tuomilehto J, Pekkanen J, Kivinen P, Kaarsalo E, Nissinen A, Karvonen MJ. Diabetes mellitus, impaired glucose tolerance and mortality among elderly men: The Finnish cohorts of the Seven Countries Study. Diabetologia 1992:35:760-765.

Stengård J, Pekkanen J, Tuomilehto J, Kivinen P, Kaarsalo E, Tamminen M, Nissinen A, Karvonen MJ. Changes in glucose tolerance among elderly Finnish men during a five-year follow-up: the Finnish Cohorts of the Seven Countries Study. Diabet Metabol 1992 (in press).

Szathmary EJE, Holt N: Hyperglycaemia in Dogrib Indians of the Northwest Territories, Canada: association with age and a centripetal distribution of body fat. Human Biol 1983;55:493-515.

Taylor HL, Jacobs DR Jr, Schucker B, Knudsen J, Leon AS, Debacker G. A questionnaire for the assesment of leisure time physical activities. J Chron Dis 1978;31:741-55.

Tuomilehto J: Primary prevention of non-insuliln-dependent diabetes mellitus: a dream or reality? In: Frontiers of diabetes research: current trends in non-insulin-dependent diabetes mellitus (eds. Alberti KGMM and Mazze R) Elsevier Science Publishers 1989;7:101-115.

Tuomilehto J, Marti B, Kartovaara L, Korhonen HJ, Pietinen P: Body fat distribution, serum lipoproteins and blood pressure in middle-aged Finnish men and women. Rev Epidem Sante Publ 1990;38:507-515.

Tuomilehto J, Wolf E: Primary prevention diabetes mellitus. Diabetes Care 1987:10:238-248.

US National Diabetes Advisory Board: Report of clinical trials subcommittee, June 3, 1991.

Wilcox C, Bailey CJ (1991): Reconsideration of inhibitory effect of metformin on intestinal glucose absorption. J Pharm Pharmacol 1991;43:120-121.

Williams OD: A framework for the quality assurance of clinical data. Clin Pharmacol Therapeutics 1979;25 (No 5, Part 2):700-2.

Wing RR, Epstein LH, Paternostro-Bayles M, Kriska A, Nowalk MP, Gooding W: Exercise in a bahavioural weight control programme for obese patients with type 2 (non-insulin-dependent) diabetes. Diabetologia 1988;31:902-909.

World Health Organization. Diabetes mellitus: report of a WHO study group. Technical Report Series 727, Geneva 1985.
